# Overexpression of *SlMADS48* Alters the Structure of Inflorescence and the Sizes of Sepal and Fruit in Tomato

**DOI:** 10.3390/plants14213259

**Published:** 2025-10-24

**Authors:** Pengyu Guo, Xin Cheng, Chuanji Xing, Zihan Gao, Jing Xue, Xiuhai Zhang, Guoping Chen, Xuqing Chen, Zongli Hu

**Affiliations:** 1 Laboratory of Molecular Biology of Tomato, College of Bioengineering, Chongqing University, Chongqing 400044, China; guopengyucqu@163.com (P.G.); chengxin599@163.com (X.C.); gzhfilm@163.com (Z.G.); chenguoping@cqu.edu.cn (G.C.); 2 Institute of Grassland, Flowers and Ecology, Beijing Academy of Agriculture and Forestry Sciences, Beijing 100097, China; 18704404680@163.com (C.X.); xuejing@baafs.net.cn (J.X.); zhangxiuhai@baafs.net.cn (X.Z.)

**Keywords:** tomato, *SlMADS48*, sepal and fruit size, inflorescence, phytohormone, RNA-Seq

## Abstract

MADS-box transcription factors play a vital role in the development of reproductive organs and fruits. However, the mechanisms by which MADS-box transcription factors participate in determining the size of organs remain incompletely understood. This study demonstrated that the overexpression of *SlMADS48* results in elongated sepals and is accompanied by an elevated gibberellin content, compared with the wild type (WT). The interaction between SlMADS48 and several proteins (SlMC, SlMBP21, SlJOINTLESS, and SlFYFL) involved in sepal development was identified. In addition, the OE*-SlMADS48* lines exhibited increased branches and total numbers of flowers. Molecular analysis revealed that SlMADS48 interacted with TM29, FUL1, FUL2, and MBP20, which are associated with inflorescence development. Moreover, SlMDS48 directly targeted the promoter of *SlTM3* via the CArG-box motif, reducing its transcript levels. Additionally, the overexpression of *SlMADS48* led to a reduction in the size of fruit, together with decreased contents of cytokinins and indole acetic acid (IAA) compared with the WT. Furthermore, SlMADS48 directly combined with the promoters of *SlcycD6;1* and *SlIAA29* in the cytokinin and auxin pathways, respectively. This research advanced our understanding of SlMADS48’s role in determining organ size and provided valuable insights into target gene selection in tomato breeding programs.

## 1. Introduction

Floral organ size is determined by the combined effects of genetic and environmental factors [1]. Recent studies have identified numerous genes involved in regulating floral organ size in tomato. For instance, the silencing of *AGL6* generated shorter petals compared with the WT [2]. Similarly, the silencing of *SlCMB1* led to elongated sepals and infinite inflorescence [3]. The silencing of *SlGLO1* produced shorter petals and male sterility [4]. SlMBP21, a regulator of cell expansion, influenced sepal size [5]. Moreover, the knockout of *MADS1* in tomato led to elongated sepals, and protein–protein interactions between SlMADS1 and SlMC was identified, illustrating the function of *SlMADS1* in flower organ size control [6]. Although these findings establish TFs as crucial regulators controlling floral organ size, the specific function of MADS-box proteins in this process requires further investigation.

Inflorescence architecture is a crucial morphological trait in flowering plants, significantly influencing the number of flowers per plant and subsequent fruit shape and yield [7,8]. Research has established that flower number and branching are determined by axillary meristem activity and developmental patterns [9]. It has been identified that TFs are key determinants of inflorescence architecture. The mutation of *tmf* (*terminating flower*), encoding an ALOG TF, resulted in the single-flower primary inflorescence phenotype in tomato [10]. Additionally, *s* (*compound inflorescence*) and *an* (*anantha*) mutations exhibited increased branching compared with a control group [11]. However, the mutation of *sft* resulted in reduced total flower number and altered inflorescence structure [12]. In *A. thaliana*, the miR172-AP2 module functions in controlling inflorescence meristem size, AP2’s transcriptional regulation by ARF3 fine-tuning shoot apical meristem (SAM) size determination [13]. In addition, the important role of MADS-box proteins in governing inflorescence development has also been identified. For example, the *simads34* mutant in *Setaria italica* exhibited an altered inflorescence structure with a wider and shorter inflorescence, illustrating its role in inflorescence determination [14]. In maize, the MADS-box genes *ZMM4*, *ZMM8*, and *ZMM14* were identified as important regulators of inflorescence development [15,16]. In tomato, the STM3-JOINTLESS2 complex and TM3/STM3-FUL2/MBP20 were also identified as important regulators of inflorescence development [17,18]. However, the role of MADS-box genes in inflorescence development remains incompletely understood.

The MADS-box gene family, widely distributed among eukaryotes, plays a crucial role in growth and development. The MADS-box transcription factor (TF) family is defined based on the sequence similarities of proteins across various eukaryotic organisms [19]. “MADS” is an initialism for the proteins MCM1, AG, DEF, and SRF, which were among the first to be studied and contain a highly conserved sequence of approximately 60 amino acids in their N-terminal regions [20]. MADS-box proteins, containing this conserved domain, have been extensively studied, and MADS-box family members have been identified in tomato (*Solanum lycopersicum*) [21], *Arabidopsis* (*Arabidopsis thaliana*) and rice (*Oryza sativa* L.) [22], soybean (*Glycine max*) [23], maize (*Zea mays* L.) [24], wheat (*Triticum aestivum* L.) [25], and apple (*Malus domestica*) [26]. In addition, the functional diversity of MADS-box TFs has been progressively uncovered, their functions encompassing phytohormone plant hormone regulation [27], abiotic stress response [28], fruit ripening [29], plant morphology [30,31], and secondary metabolism [32,33], among others.

Tomato (*Solanum lycopersicum*) is a fleshy fruit-bearing crop globally recognized for its high nutritional and commercial value. Tomato yield is closely correlated with fruit size. Previous research has established that fruit size regulation primarily involves hormonal mechanisms. For example, the overexpression of tomato *SlCDF4* (*Cycling DOF Factor 4*) under the specific promoter of *PPC2* increased gibberellin GA_4_ content, resulting in larger fruit size [34]. The overexpression of a CK-inactivating enzyme gene, *AtCKX2* (*Arabidopsis thaliana*), under the control of the *Tfm7* promoter in tomato, altered the endogenous cytokinin content, leading to smaller fruit [35]. In addition, the crosstalk between different plant hormones influences the determination of fruit size. The tomato *amiARF5* transgenic lines showed a phenotype of reduced fruit size compared with the WT, involving both auxin and gibberellin signaling pathways [36]. Previous research also illustrated that MADS-box TFs functioned in the determination of fruit sizes. For instance, the heterologous overexpression of *VvMADS39* (*Vitis vinifera*) in tomato led to reduced fruit size [37]. In *Arabidopsis*, the loss-of-function mutant gordita, encoded by a B-sister MADS-box gene, exhibited larger fruit compared with the wild type, illustrating its role in fruit growth regulation [38]. Similarly, the overexpression of *CmFYF* (*Cucumis melo* L.) generated fruit smaller in size compared with the wild type [39]. However, how the MADS-box TF influences fruit size in tomato is still not fully understood.

During our previous systematic bioinformatic analysis of the tomato MADS-box gene family, we noted that the biological function of MADS48 remained uncharacterized, as also indicated by the available literature. Furthermore, the preliminary functional characterization of several MADS-box genes suggested a potential role for MADS48 in floral organ development. These findings prompted us to select MADS48 for in-depth functional analysis, by integrating molecular, anatomical, and physiological approaches. The overexpression of *SlMADS48* led to elongated sepals together with increased GA_1_ content. Moreover, the protein interaction between SlMADS48 and JOINTLESS, MBP21, MC and FYFL, which were involved in sepal development, was identified. In addition, the OE*-SlMADS48* lines also exhibited altered inflorescence with increases in the number of branches and flowers. SlMADS48 also demonstrated interaction capability with TM29, FUL1, FUL2, and MBP20. Furthermore, SlMADS48 bound to the CArG-box motif in the promoter of *SlTM3* and inhibited its transcript level. We also found that the overexpression of *SlMADS48* led to smaller fruit and the decreased content of endogenous iP7G, tZR, tZRMP, and IAA. *SlcycD6;1* and *SlIAA29* were identified as the downstream target genes of SlMADS48. This study enhances our understanding of how tomato MADS-box TFs regulate floral organ development and determine fruit size. It establishes a theoretical foundation for investigating MADS-box genes in tomato and other plants while providing candidate genes for tomato breeding.

## 2. Results

### 2.1. Characterization and Tissue Expression Pattern Analysis of the SlMADS48 Gene

**Figure 1:**
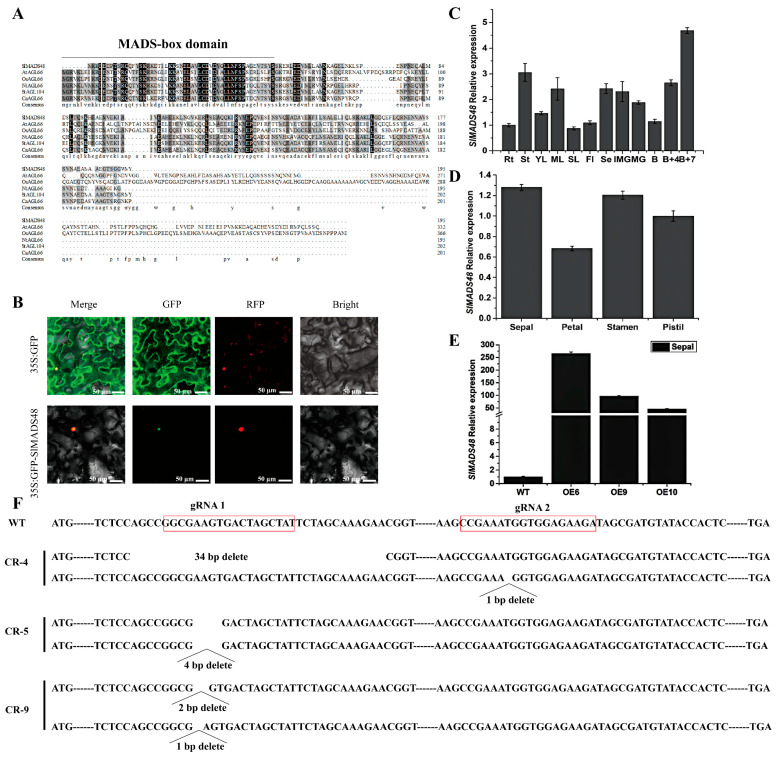
**The protein alignment, subcellular localization, and expression patterns of SlMADS48, and the obtaining of *SlMADS48*-overexpressed (OE) and *SlMADS48*-CRISPR/Cas9 mutant (CR) lines.** (**A**) Protein multiple sequence alignment between SlMADS48 and homologous genes in other species: *At, Arabidopsis thaliana*; *Ca, Capsicum annuum*; *Nt, Nicotiana tabacum*; *Os, Oryza sativa*; *St, Solanum tuberosum*. (**B**) The subcellular localization of SlMADS48. (**C**) The expression pattern of *SlMADS48* in different organs, including Rt (root), St (stem), YL (young leaves), ML (mature leaves), SL (senescent leaves), Fl (flower), Se (sepal), IMG (immature green fruit) and MG (mature green fruit) at B (breaker), B+4 (4 days after breaker), and B+7 (7 days after breaker). (**D**) The expression pattern of *SlMADS48* in four-whorl floral organs, sepals, petals, stamens, and pistils. Values are means ± standard errors. (**E**) The selection of three independent SlMADS48-overexpressed lines with higher transcript levels of *SlMADS48*, via qRT-PCR, using sepals of candidate overexpressed transgenic lines. (**F**) The genotyping of knockout mutant alleles.

In accordance with our previous research [21], a tomato MADS-box gene, designated *SlMADS48*, was isolated from WT tomato (*Solanum lycopersicum*) flower. Located on chromosome 7, *SlMADS48* encoded a protein of 195 amino acids, with a molecular weight of 20.88 kD and an isoelectric point of 4.86. Protein sequence analysis indicated that a conversed MADS-box domain was present in the N-terminal of SlMADS48 (Figure S1A) and three-dimensional structure was shown in Figure S1B. The multiple sequence analysis of SlMADS48 and homologous proteins in other species further demonstrated that SlMADS48 was a typical MADS-box TF (Figure 1A). Subcellular localization assays revealed that SlMADS48 was predominantly localized in the nucleus (Figure 1B). Then, the prediction of *cis*-acting regulatory elements in its promoter using PlantCARE [40] included phytohormone responses (such as auxin, gibberellin, abscisic acid, salicylic acid, and MeJA) and abiotic stress (such as drought), indicating that SlMADS48 may play an essential role in responding to phytohormones and abiotic stresses (Table S2). To further understand the biological functions of *SlMADS48*, we performed a tissue expression pattern analysis of *SlMADS48* in the WT. As shown in Figure 1C, *SlMADS48* transcripts were successfully detected in all tissues, which showed a relatively higher abundance in B+7 (7 days after breaker) fruit and stems and B+4 (4 days after breaker) fruit, sepals, and mature leaves, and exhibited the lowest expression level in the senescent leaves. Moreover, *SlMADS48* transcripts were highly accumulated in the sepals, stamens, and pistils of four-whorl flower organs in WT tomato (Figure 1D).

### 2.2. Overexpression of SlMADS48 Generated Elongated Sepals

To further explore the biological function of *SlMADS48* in tomato, *SlMADS48*-overexpressed lines (designated OE-*SlMADS48*) and *SlMADS48*-CRISPR-Cas 9 (called CR-SlMADS48) mutant lines were generated. Based on the results of the qRT-PCR assay, three independent *SlMADS48*-overexpressed lines, OE-6/OE-9/OE-10, with evidently higher transcript levels of *SlMADS48* compared with the WT were selected for further research (Figure 1E and Figure S1C). In addition, three independent mutant lines, CR*-SlMADS48*-4, CR*-SlMADS48*-5 and CR*-SlMADS48*-9, were also obtained, and the genotype is shown in Figure 1F.

**Figure 2:**
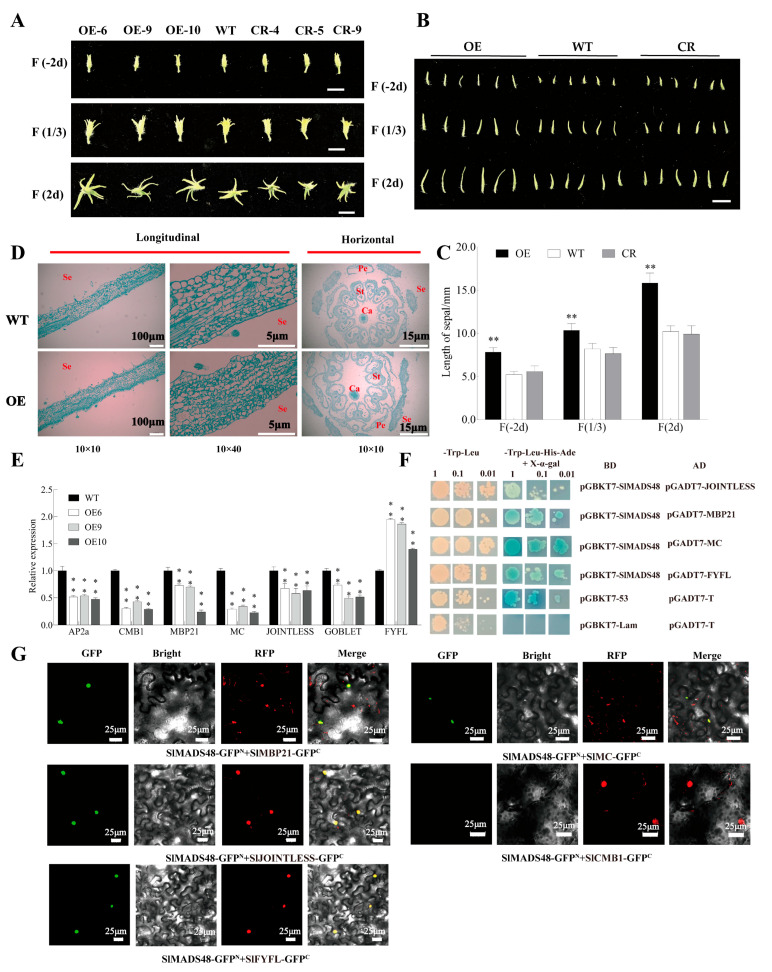
**The overexpression of *SlMADS48* alters the size of sepals in tomato.** (**A**,**B**) The phenotype of sepals of WT, OE, and CR lines at F (−2 d), F (1/3), and F (2 d). (**C**) A comparison of sepal length between WT, OE, and CR lines at different stages. Data are the mean ± SE of ten biological replicates. ** indicates significant difference, Student’s *t*-test *p* < 0.01. (**D**) Anatomical analysis of sepals and flowers by paraffin section between WT and OE lines. (**E**) The transcript level of genes involved in the development of sepals, including *AP2a*, *CMB1*, *MBP21*, *MC*, *JOINTLESS*, *GOBLET*, and *FYFL*. Data are the mean ± SE of three biological replicates. ** indicates significant difference, Student’s *t*-test *p* < 0.01. (**F**,**G**) The identification and confirmation of protein–protein interaction between SlMADS48 and other proteins involved in sepal development. In Y2H (**F**), the pGBKT7-53 + pGADT7-T was set as the positive control and pGBKT7-Lam + pGADT7-T was set as the negative control. The leftmost column represents the bacterial culture at OD_600_ = 1, with subsequent columns containing 10-fold and 100-fold serial dilutions for spot assay. Photographs were taken after 5 days of inverted incubation at 30 °C. In BiFC (**G**), the SlMADS48-GFP^N^ + SlCMB1-GFP^C^, which did not interact, was used as the negative control. The injected tobacco plants were kept in darkness for 2 days post-injection, followed by imaging using a confocal laser scanning microscope.

During the development of the flowers, we found that OE*-SlMADS48* lines exhibited longer sepals compared with the WT, while no significant difference was observed between the WT and CR-*SlMADS48* lines (Figure 2A,B). Further, we also measured the sepal length of OE*-SlMADS48*, CR*-SlMADS48*, and the WT at different stages of flower development [sepal at −4 days post-anthesis (dpa), sepal at anthesis, and sepal at 4 days post-anthesis (dpa)], and the results also confirmed this phenotype (Figure 2C). Subsequently, to further elucidate the underlying causes of the observed phenotypes, anatomical analyses of sepals and whole flower buds at −2 days of OE-*SlMADS48* lines and WT were performed (Figure 2D).

### 2.3. SlMADS48 Interacted with MBP21, JOINTLESS, MC, and FYFL

Previous research illustrated that *AP2a*, *SlCMB1*, *SlMBP21*, *MC*, *JOINTLESS*, *GOBLET*, and *SlFYFL*, in tomato, participated in the sepals’ developmental regulation [3,5,41,42,43,44,45]. To explore the relationship between the abovementioned phenotype and these genes, the transcript levels of the genes in OE*-SlMADS48* and WT sepals were determined. The qRT-PCR results revealed that the mRNA abundance of *AP2a*, *SlCMB1*, *SlMBP21*, *MC*, *JOINTLESS*, and *GOBLET* decreased, while *SlFYFL* increased in the sepals of OE*-SlMADS48* lines (Figure 2E). These results demonstrate that the overexpression of *SlMADS48* has an influence on the transcript abundance of genes involved in the development of sepals.

Previous studies demonstrated that MADS-box proteins function in the regulation of plant growth and development by forming homodimers, heterodimers, or higher protein complexes [46,47,48,49]. Therefore, we speculated that SlMADS48 potentially interacts with sepal-development-related proteins to jointly regulate tomato sepal development. In order to verify this hypothesis, extensive Y2H assays were performed. The CDS of *SlMADS48* was ligated to the pGBKT7 vector, as the bait, and the CDS of *SlAP2a*, *SlCMB1*, *SlMBP21*, *MC*, *JOINTLESS*, and *SlFYFL* was amplified and cloned into the pGADT7 vector as the prey, respectively. The experiments revealed that pGBKT7-SlMADS48 lacked self-activation activity. The Y2H experiment showed that JOINTLESS, SlMBP21, MC, and SlFYFL physically interacted with SlMADS48, respectively, while no protein–protein interaction was observed between SlMADS48 and CMB1 or AP2a (Figure 2F and Figure S2A). Furthermore, the BiFC results confirmed interactions between SlMADS48 and JOINTLESS, SlMBP21, MC, and SlFYFL (Figure 2G), indicating that SlMADS48 regulated the development of sepals, possibly by interacting with these four proteins.

### 2.4. Transcriptome Analysis of Sepals Between OE-SlMADS48 and WT

To further explore the molecular mechanism of *SlMADS48* in regulating sepal size, a global comparison of transcription accumulation and DEGs of −2-dpa sepals between WT and OE*-SlMADS48* was performed using RNA-seq. (Because there was no significant difference, the sepals from WT and OE-*SlMADS48* lines were collected to perform the RNA-seq.) The sequencing generated more than 44 million clean reads in each of the four cDNA libraries, resulting in a comprehensive dataset of over 6.5 Gb of sequence data per sample (Table S3). The Pearson’s correlation coefficient indicated an enhanced correlation between the four samples (Figure S2B). In addition, a total of 22,707 genes were detected in the WT and OE*-SlMADS48*, with 944 genes exclusively detected in the WT and 896 genes in OE*-SlMADS48* lines (Figure S2C). The analysis revealed that 1359 differentially expressed genes (DEGs) were enriched in OE*-SlMADS48* lines relative to WT plants, including 817 upregulated and 542 downregulated genes (Figure S2D). To investigate the accurate functions of the DEGs between OE*-SlMADS48* sepals and WT sepals, the DEGs were categorized into three categories: biological process (BP), cellular component (CC), and molecular function (MF). For BP, the DEGs were primarily enriched in metabolic and cellular processes. For MF, DEGs were predominantly associated with catalytic activity. For CC, the majority of DEGs were connected to cells, organelles, and membranes (Figure S2E). The KEGG (Kyoto Encyclopedia of Genes and Genomes) analyses clustered the DEGs, with the top three categories being ribosome, biosynthesis of amino acids, and phenylpropanoid biosynthesis (Figure S2F).

### 2.5. Elongated Sepals Possibly Associated with Increased Gibberellin

**Figure 3:**
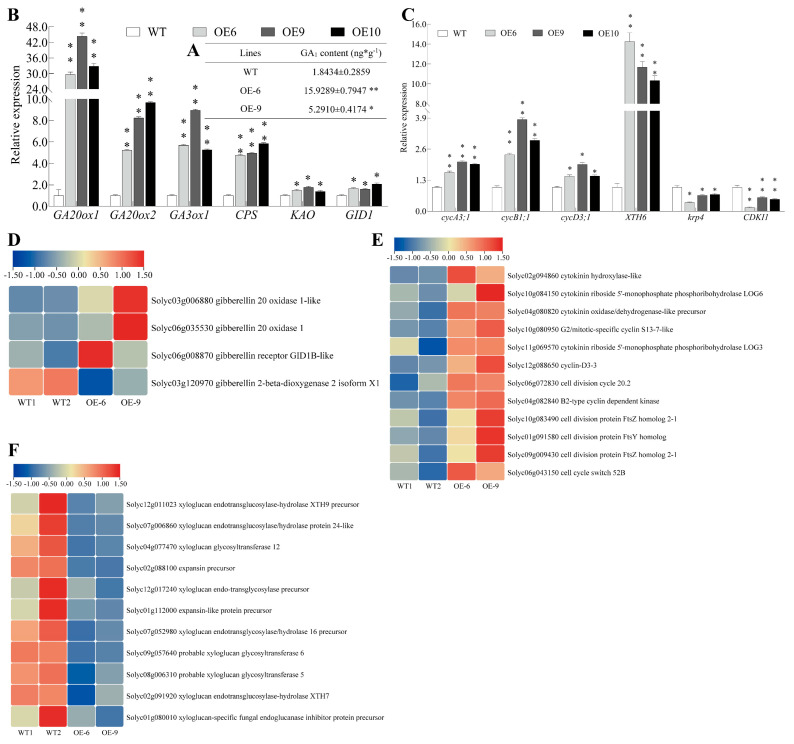
**The overexpression of *SlMADS48* influences the content of endogenous gibberellin in sepals and the transcript levels of genes involved in gibberellin and cell division.** (**A**) Examination of endogenous gibberellin in sepals of WT and OE lines. Data are the mean ± SE of three biological replicates. * indicates significant difference, Student’s *t*-test *p* < 0.05, ** *p* < 0.01. (**B**) Transcript level of genes involved in the gibberellin pathway, including biosynthesis genes such as *GA20ox1*, *GA20ox2*, *GA3ox1*, *CPS*, and *KAO*, and the signaling transduction gene *GID1*. Data are the mean ± SE. * indicates significant difference, Student’s *t*-test *p* < 0.05, ** *p* < 0.01. (**C**) Detection of expression level of genes involved in the cell cycle, including *cycA3;1*, *cycB1;1*, *cycD3;1*, *krp4*, and *CDKI1*. Data are the mean ± SE. * indicates significant difference, Student’s *t*-test *p* < 0.05, ** *p* < 0.01. (**D**–**F**) Heatmap of DEGs (differentially expressed genes) from RNA-seq data involved in the cell cycle, cell division (**D**), the gibberellin pathway (**E**), and cell expansion (**F**).

Changes in endogenous hormone levels are crucial for the development of plant organs. Based on the observed sepal phenotype, we detected the content of endogenous gibberellin, which revealed that the sepals of OE*-SlMADS48* lines showed an evidently increased accumulation of GA_1_ compared with the WT (Figure 3A). Subsequently, a qRT-PCR assay indicated substantial changes in the transcript level of genes involved in the gibberellin pathway, which exhibited dramatic alterations. The expression levels of *GA20ox1*, *GA20ox2*, *GA3ox2*, *CPS*, and *KAO* in the OE lines, which are involved in bioactive gibberellin biosynthesis, and GID1, which is involved in signal transduction, were enhanced significantly compared with the WT (Figure 3B). Organ changes frequently correlate with changes in cell number. Furthermore, we found that the mRNA abundance of the cell cycle inhibitors *krp4* and *CDKI1* were significantly lower than those in the WT, while the expression levels of the cell-cycle-related genes *cycA3;1*, *cycB1;1*, *cycD3;1*, and *XTH6* were markedly higher than those in the WT (Figure 3C). In addition, using RNA-seq data, we isolated the DEGs involved in gibberellin (Figure 3D) and cell division (Figure 3E), and some genes were significantly upregulated. It is worth noting that the transcript levels of genes involved in cell expansion in OE lines were substantially inhibited (Figure 3F).

### 2.6. Overexpression of SlMADS48 Alters Inflorescence Structure

**Figure 4:**
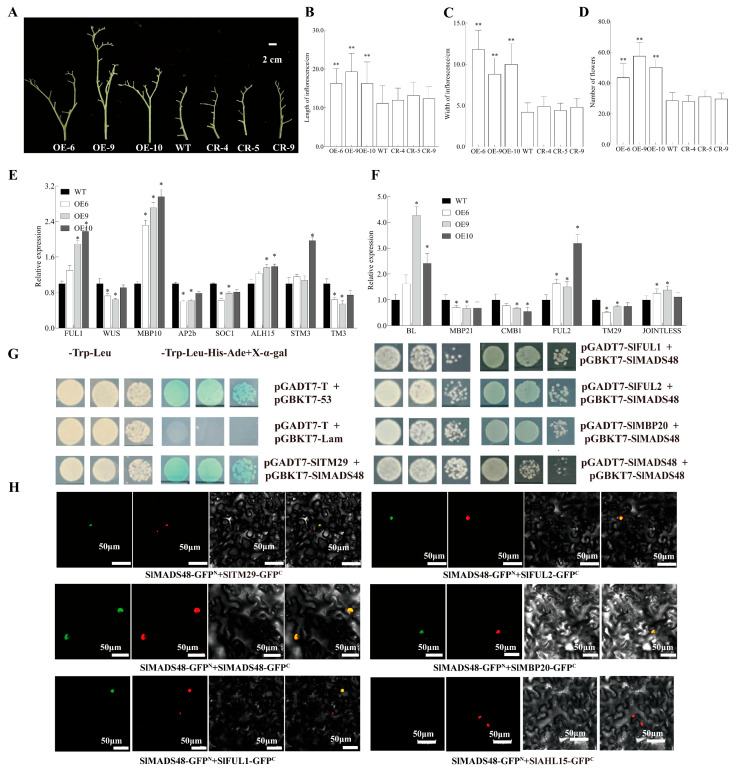
**The overexpression of *SlMADS48* alters the structure of inflorescence.** (**A**) Phenotype of inflorescence for WT, OE, and CR lines. Bar: 2 cm. (**B**–**D**) Statistics of the length and width of inflorescence and the number of flowers on the whole plant. Data are the mean ± SE. ** indicates significant difference, Student’s *t*-test *p* < 0.01. (**E**,**F**) Transcript levels of genes involved in inflorescence development. Data are the mean ± SE. * indicates significant difference, Student’s *t*-test *p* < 0.05. (**G**,**H**) Identification and confirmation of protein–protein interaction between SlMADS48 and other proteins involved in inflorescence development. In Y2H, pGBKT7-53 + pGADT7-T was set as the positive control and pGBKT7-Lam + pGADT7-T was set as the negative control. In BiFC, SlMADS48-GFP^N^ + SlAHL15-GFP^C^, which does not interact with yeast, was used as the negative control.

The OE*-SlMADS48* lines exhibited an altered inflorescence structure, characterized by increased branching (Figure 4A). In addition, these lines also exhibited increased inflorescence length, width, and number of flowers per plant compared with the WT (Figure 4B–D). However, no significant differences were observed between the WT and SlMADS48-CR lines (Figure 4A–D). Subsequently, for exploring the regulatory mechanism underlying this phenotype, we detected the transcript level of genes related to inflorescence development in the WT and OE-*SlMADS48* lines, revealing varying degrees of upregulation or downregulation of genes involved in inflorescence development (Figure 4E,F).

**Figure 5:**
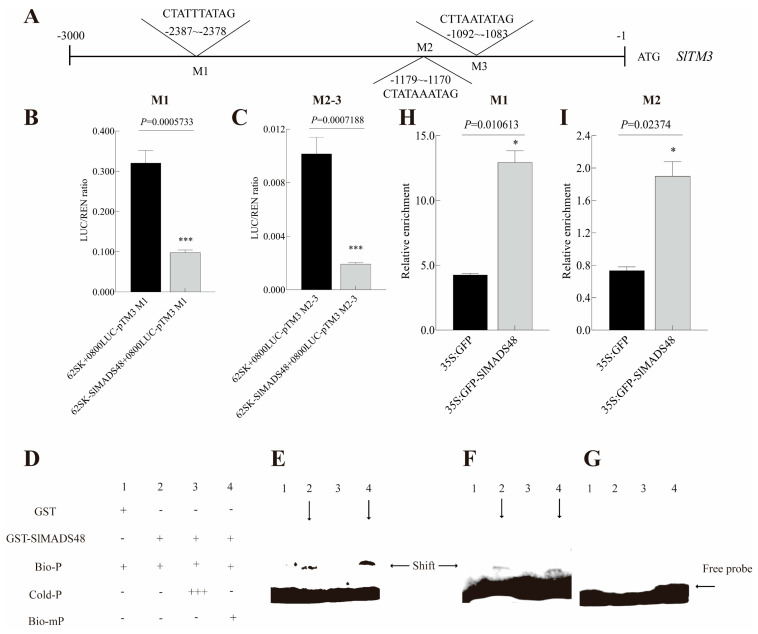
**SlMADS48 directly targeted the promoter of *SlTM3*.** (**A**) The location of 3 CArG-box motifs in the promoter of *SlTM3* region before ATG. (**B**,**C**) The Dual-LUC assay results of SlMASD48 on M1 (**D**) and M2-3 (**E**) in the promoter of *SlTM3*. (**D**) A schematic diagram of the EMSA experiment. (**E**–**G**) The EMSA assay results of CArG-box motifs, M1 (**E**), M2 (**F**), and M3 (**G**). (**H**,**I**) The ChIP-qPCR results of M1 (**H**) and M2 (**I**) CArG-box motif. Data are the mean ± SE. * indicates significant difference, Student’s *t*-test *p* < 0.05, *** *p* < 0.001.

To further elucidate the molecular regulatory mechanisms underlying the observed phenotype, Y2H assays were performed using SlMADS48 as the bait to screen the interacted proteins involved in tomato inflorescence development. The results identified that SlMADS48 interacted with TM29, FUL1, FUL2, and MBP20 (Figure 4G), while no protein–protein interaction between SlMADS48 and FA, WUS, and AHL15 was observed (Figure S3A). Subsequently, the BiFC experiment was employed to confirm the results (Figure 4H). A Dual-LUC assay was used to identify potential target genes or genes downstream of SlMADS48 involved in inflorescence structure alteration. The ratio of LUC/REN was evidently inhibited in the experimental group (Figure S4A and Figure 5A–C), illustrating that SlMADS48 bound to the promoter of *SlTM3* and inhibited its transcription. EMSA identified that SlMADS48 bound specifically to M1 and M2 of the CArG box rather than M3 (Figure 5D–G), which was confirmed by ChIP-qPCR analysis (Figure 5H,I).

### 2.7. Overexpression of SlMADS48 Changes Fruit Size with Altered Endogenous Hormones

**Figure 6:**
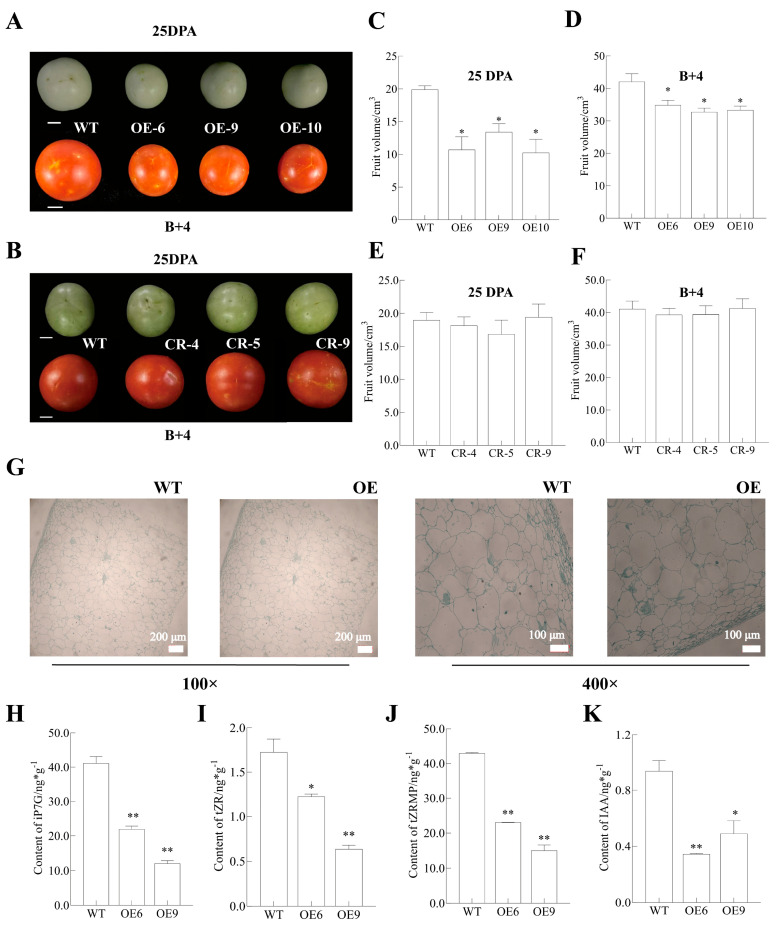
**The overexpression of *SlMADS48* has an effect on fruit size determination and the accumulation of endogenous auxin and cytokinin.** (**A**,**B**) The phenotype of fruit from WT, OE, and CR lines at 25DPA and B+4 stage. Bar: 1 cm. (**C**–**F**) A comparison of fruit volume between WT vs. OE lines and WT vs. CR lines of 25DPA at the B+4 stage. Data are the mean ± SE. * indicates significant difference, Student’s *t*-test *p* < 0.05. (**G**) Anatomical analysis of 25DPA fruit between WT and OE lines. (**H**–**K**) Detection of endogenous plant hormone content in 25DPA fruit, including iP7G, tZR, tZRMP, and IAA, in WT and OE lines. Data are the mean ± SE. * indicates significant difference, Student’s *t*-test *p* < 0.05, ** *p* < 0.01.

In addition to the alteration of sepals and inflorescence, the overexpression of *SlMADS48* also modified the size of the fruit, but there was no significant difference between the WT and CR-*SlMADS48* lines (Figure 6A,B). The analysis of fruits from the WT and OE and CR lines at 25 DPA and B+4 revealed that SlMADS48-OE lines exhibited reduced fruit volume compared with the WT (Figure 6C,D). Furthermore, the fruit weight, height, and diameter of the SlMADS48-OE lines were decreased compared with the WT (Figure S5A–F). In contrast, SlMADS48-CR lines demonstrated no significant differences in fruit parameters compared with the WT, including volume, diameter, weight, and height (Figure 6E,F and Figure S5G–L).

Based on the phenotype exhibited by the OE-*SlMADS48* lines, the 25DPA fruit of the WT and OE-*SlMADS48* lines were collected and an anatomical analysis was performed (Figure 6G). To further clarify the possible molecular mechanism, the endogenous hormone content was quantified in both the WT and OE*-SlMADS48* lines. The results exhibited a decreased level of the cytokinin components tZRMP, tZR, and iP7G (Figure 6H–J), as well as auxin IAA content in transgenic lines compared with the WT (Figure 6K). Subsequently, the qRT-PCR results revealed that the transcript levels of the genes involved in auxin biosynthesis, auxin transport, and auxin response were downregulated to varying degrees (Figure S5M). Moreover, the transcript levels of *cycA3;1*, *cycB1;1*, and *cycD3;1*—the cell cycle genes—were inhibited significantly, and *krp4* and *CDKA1*, the inhibitors of cell division, were evidently enhanced (Figure S5N). In addition, genes involved in cell expansion/elongation and cell wall metabolism exhibited lower mRNA abundance in the fruits from OE*-SlMADS48* lines compared with the WT (Figure S5O).

### 2.8. RNA-Seq of 25DPA Fruit Between WT and OE-SlMADS48

To further explore the molecular mechanism of *SlMADS48* overexpression in terms of fruit size, an RNA-seq analysis was performed on 25-DPA fruit samples from both the WT and OE-*SlMADS48* lines. The sequencing generated more than 45 million clean reads in each of the four cDNA libraries, resulting in a comprehensive dataset of over 6.75 Gb of sequence data for per sample (Table S4). A Pearson correlation coefficient analysis revealed a strong correlation between the WT and OE*-SlMADS48* lines (Figure S6A). In addition, 16,305 genes were detected in both the WT and transgenic lines (Figure S6B), with 2275 differentially expressed genes identified, comprising 971 upregulated and 1304 downregulated genes (Figure S6C). A Gene Ontology (GO) analysis was performed to categorize the DEGs into three major functional categories. The analysis revealed that the DEGs were primarily enriched in BP terms associated with the cellular glucan metabolic process, glucan metabolic process, and cellular polysaccharide metabolic process. In terms of MF, most of the DEGs were linked to DNA-binding transcription factor activity, xyloglucan/xyloglucosyl transferase activity, and transcription regulator activity. Regarding CC, the majority of the DEGs were associated with the cell wall, external encapsulating structure, and apoplast (Figure S6D). Similarly, the Kyoto Encyclopedia of Genes and Genomes (KEGG) enrichment analysis demonstrated the involvement of DEGs in pathways such as plant hormone signal transduction (Figure S6E).

### 2.9. Analyses of DEGs in 25DPA Fruit Between WT and OE-SlMADS48 Lines and Validation by qRT-PCR

**Figure 7:**
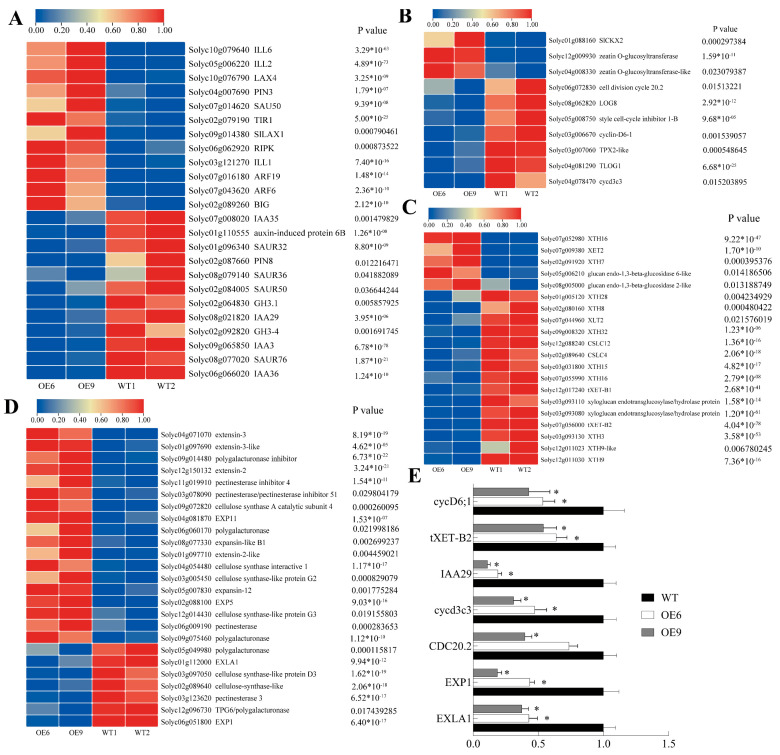
**The isolation of DEGs involved in the auxin pathway, cell division, and cell wall development, and the validation of RNA-seq data via qRT-PCR assay.** (**A**) The DEGs involved in the auxin pathway. (**B**) The DEGs involved in cell division. (**C**,**D**) The DEGs involved in cell wall development. (**E**) The validation of RNA-seq data via qRT-PCR assay. Data are the mean ± SE. * indicates significant difference, Student’s *t*-test *p* < 0.05.

Subsequently, based on the phenotype difference between OE*-SlMADS48* and WT and the DEG enrichment pattern, DEGs related to phytohormones and cell development were isolated from RNA-seq data and visualized using TBtools. DEGs were involved in the auxin pathway, where *GH3.1* (auxin biosynthesis), *PIN8* (auxin transport), *IAA3*, *IAA36*, and *SAUR50* (auxin response) were significantly downregulated, while *ARF6*, *ARF19*, *LAX1*, and *TIR1* were upregulated in fruits of SlMADS48-OE lines (Figure 7A). Moreover, compared with the WT, the transcript levels of *TLOG1*, *CYCD6-1*, *CYCD3-C3* and *CDC20-2*, which are involved in the cytokinin pathway, were inhibited significantly (Figure 7B). The correlation of fruit size with cell wall development was evident, as multiple XTH genes were significantly repressed in the fruits of SlMADS48-OE lines (Figure 7C). While the expression of *EXP11*, *EXP5*, and polygalacturonase increased in transgenic lines, the expression of *EXLA1*, *EXP1*, and pectinesterase 3 was notably inhibited (Figure 7D). The qRT-PCR validation of selected genes confirmed consistency with RNA-seq data (Figure 7E).

### 2.10. SlMADS48 Directly Targeted SlIAA29 and SlcycD6;1

**Figure 8:**
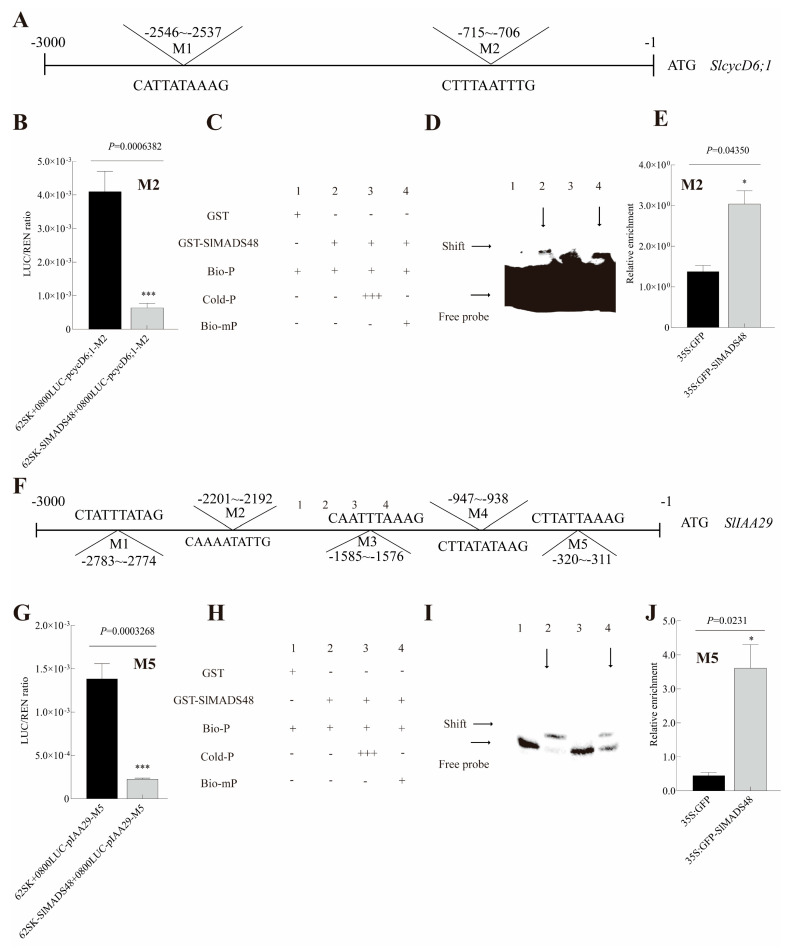
**SlMADS48 bound the promoter of *SlcycD6;1* and *SlIAA29* via the CArG motif.** (**A**) The location of two CArG-box motifs in the promoter region before the start codon of *SlcycD6;1*. (**B**) The Dual-LUC assay results of SlMASD48 on the M2 motif in the promoter of *SlcycD6;1*. (**C**,**D**) A schematic diagram of the EMSA experiment and the EMSA results for SlAMDS48 on the M2 motif in the promoter of *SlcycD6;1*. (**E**) The ChIP-qPCR results using *35S:SlMADS48-GFP* and *35S:GFP* plants with the anti-GFP antibody, targeting M2 in the promoter of *SlcycD6;1*. (**F**) The location of 5 CArG-box motifs in the promoter region before the start codon of *SlIAA29*. (**G**) The Dual-LUC assay results for SlMASD48 on M5 (M). (**H**,**I**) A schematic diagram of the EMSA experiment and the EMSA results for SlAMDS48 in the M5 motif in the promoter of *SlIAA29*. (**J**) The ChIP-qPCR results using *35S:SlMADS48-GFP* and *35S:GFP* plants with the anti-GFP antibody, targeting M5 in the promoter of *SlIAA29*. Data are the mean ± SE. * indicates significant difference, Student’s *t*-test *p* < 0.05, *** *p* < 0.001.

In conjunction with the reduced fruit size and decreased content of cytokinin and IAA, the evidence demonstrated that SlMADS48 possibly regulates fruit size through the auxin and cytokinin pathways. To further unravel the molecular mechanism, we performed a Dual-LUC assay for identifying potential downstream target genes. The results showed that SlMADS48 possessed the ability to bind to the promoter and inhibited the transcription of *SlcycD6;1* and *SlIAA29* via the M2 CArG-box motif in the promoter of *SlcycD6;1* (Figure 8A and Figure S7A,B) and the M5 CArG-box motif in the promoter of *SlIAA29* (Figure 8F,G and Figure S7C–G). EMSA subsequently confirmed the binding of SlMADS48 to both promoters (Figure 8C,D,H,I). In addition, ChIP-qPCR assays confirmed these interactions (Figure 8E,J). These findings demonstrate that SlMADS48 targets the promoters of *SlcycD6;1* and *SlIAA29* via CArG box motifs, thereby participating in regulating fruit size through the auxin and cytokinin pathways.

## 3. Discussion

### 3.1. Longer Sepals of OE-SlMADS48 Lines Were Possibly Caused by Increased Gibberellin and Interaction with Proteins Involved in Sepal Development

In plants, organ elongation strongly correlates with changes in endogenous GA accumulation and the expression of GA pathway genes. For instance, in maize leaf base development, active GA accumulation determines the division zone size [50]. The overexpression of SlCRCa, which functions in the feedback regulation of GA biosynthesis, results in reduced petal, stamen, and fruit size in tomato [51]. Longer leaves in wild rice accumulate more GA, with enhanced cell division leading to increased leaf length. The downstream genes *OsGRF7* and *OsGRF8* regulate cell division, thereby affecting rice leaf length [52]. In tomato *dsp* mutants, the decreased accumulation of gibberellin influences the morphology of sepals [53]. In the present study, the overexpression of *SlMADS48* resulted in longer sepals compared with the WT, with increased GA1 content. qRT-PCR analysis revealed elevated transcript levels of GA biosynthesis genes, including GA20ox1, GA20ox2, GA3ox1, and CPS, corresponding to increased GA1 levels. It was suggested that the overexpression of AtGA20ox affects hypocotyl length, leaf size, and stem height at the cellular level [54]. Moreover, the qRT-PCR results showed that the mRNA abundance of *cycA3;1*, *cycB1;1*, and *cycD3;1* [55,56], which function as positive regulators of the cell cycle, were obviously upregulated, while the cell cycle inhibitors *krp4* [57] and *CDKI1* were significantly repressed in the OE*-SlMADS48* lines. In addition, the RNA-seq data showed that the transcripts of genes related to cell cycle and cell division were evidently enhanced in OE*-SlMADS48* lines compared with the WT (Figure 3E). Moreover, the genes involved in the biosynthesis of gibberellin were upregulated in the sepals of OE-*SlMADS48* lines, while the genes involved in the degradation of gibberellin were significantly downregulated (Figure 3D). These results indicate that the overexpression of SlMADS48 leads to the enhanced accumulation of gibberellin and promotes cell division, further leading to elongated sepals.

Previous research has identified that the MADS-box transcription factor plays a vital role in the development of sepals. For instance, the reduced expression of tomato *MC* generates larger sepals compared with the WT, illustrating its important role in sepal development [43]. The overexpression of *SlFYFL* driven by the 35S promoter results in longer sepals compared with the WT [42], indicating its function in sepal development. Similarly, it was reported that tomato *SlMBP21* functions negatively in the determination of sepal size [5]. The loss function of the *JOINTLESS j-2* also leads to the longer-sepal phenotype, demonstrating that *JOINTLESS* functions in tomato sepal formation [58]. In addition, accumulating evidence indicates that the MADS-box protein enables interactions with other proteins in vivo and in vitro [47], demonstrating that MADS-box transcription factors potentially function through dimerization. To explore whether SlMADS48 participates in regulating sepal development by interacting with these TFs, we performed Y2H assays using SlMADS48 as a bait protein. The results identified protein–protein interactions between SlMADS48 and MBP21, MC, JOINTLESS, and FYFL. Similarly, we performed a BiFC assay to confirm these interactions, yielding positive results. Collectively, these findings verify that SlMADS48 participates in regulating sepal development, possibly by interacting with the proteins involved in sepal development.

### 3.2. SlMADS48 Functions in Inflorescence Development by Interacting with FUL1, FUL2, MBP20, and TM29, and Directly Targeting SlTM3

In tomato, the structure of inflorescence is a crucial agronomic trait closely related to the number of flowers and the final yield [59]. The formation and development of inflorescence in tomato is complex and governed by multiple genetic factors [60,61]. To date, numerous studies have demonstrated that MADS TFs contribute to inflorescence development. For example, the tomato MADS-box transcription factor FUL2 and MBP20 function in vegetative-to-reproductive transition and as a repressor in inflorescence branching by inducing floral meristem (FM) maturation [62]. Moreover, STM3 not only interacts with FUL1, but also binds to the promoter of *FUL1*, further regulating inflorescence branching in tomato [63]. Notably, FUL1 possesses the ability to bind to the promoter of *TM3* [62]. *TM29*, the homologous gene of *SEP1*, *2*, and *3* (*SEPALLATA1*, *2*, and *3*) in *Arabidopsis*, plays a role in floral meristem identity in tomato. The silencing of *TM29* generates morphogenetic alterations in inflorescence [64]. In the present study, based on the phenotype alteration of the inflorescence that the OE-*SlMADS48* lines exhibited, we hypothesized that SlMADS48 could participate in the regulation of inflorescence development by interacting with these proteins. Further exploration (via Y2H and BiFC assays) found that SlMADS48 possessed the ability to interact with FUL1, FUL2, MBP20, and TM29. These results suggest that SlMADS48 participates in inflorescence development, possibly via the interaction with these TFs at the protein level.

In addition, the loss-function mutant *tm3* exhibited altered inflorescence compared with the WT. Moreover, TM3 shared some common downstream target genes with FUL1, including genes in the cytokinin pathway, further influencing inflorescence development [18]. Here, using a combination of phenotype observations, previous research, and the isolation of possible downstream genes, we found that SlMADS48 was able to target the CArG motifs in the promoter of *SlTM3* through the Dual-LUC, EMSA, and ChIP-qPCR assays, demonstrating that SlMADS48 participates in inflorescence development, possibly by targeting *SlTM3*.

### 3.3. SlMADS48 Directly Targets SlIAA29 and SlcycD6;1, Further Influencing Fruit Size via Auxin and Cytokinin Pathways

Tomato, a vegetable crop widely cultivated for its high nutritional and economic value, serves as a model system for studying the regulatory mechanisms governing fruit size. Research has identified multiple pathways controlling tomato fruit size, including TF regulation, phytohormone regulation, epigenetic regulation, and environmental factors. For instance, the overexpression of *NOR-like 1* reduces tomato size by directly binding to the promoter and inhibiting the transcription of *SlGRAS2* and *SlFW11.3* [65]. The endogenous phytohormones auxin and cytokinin play crucial roles in determining fruit size. In jujube (*Ziziphus jujuba*), ZjWRKY23 and ZjWRKY40 regulate fruit size by targeting and inhibiting *ZjCKX5* transcription through the cytokinin pathway [66]. Grinding-induced cytokinin accumulation leads to tomato enlargement through the cytokinin pathway [67]. It has been found that the larger size of “Grand Longfeng” (GLF) in comparison to that of “Longfeng” (LF) apple (*Malus domestica*) was caused by increased auxin content, together with the upregulated expression of *MdTAR1* and *MdYUCCA6* [68]. The overexpression of *CsMYB77* (*Citrus sinensis*) generated reduced fruit size in citrus, together with a decreased auxin content [69]. A notable feature of the maize *defective endosperm 18* (*de18*) mutant is its decreased kernel size due to the impaired accumulation of auxin [70]. These results indicate that auxin and cytokinin play a vital role in fruit size determination. In the present study, the overexpression of *SlMADS48* generated the decreased-size fruit phenotype. The detection of endogenous plant hormones showed that the fruit of *OE-SlMADS48* lines possessed lower levels of cytokinin-associated iP7G/tZR/tZRMP and auxin-associated IAA (Figure 6H–K). In addition, the qRT-PCR assay demonstrated that the transcription levels of some genes involved in auxin biosynthesis, auxin transport, and auxin response were considerably inhibited (Figure S5M). Moreover, the expression levels of *cycA3;1*, *cycB1;3*, and *cycD3;1*, which are positive regulators of the cell cycle, were downregulated significantly, and the expression levels of *CDKI1* and *krp4*, inhibitors of cell division, were enhanced compared with the WT (Figure S5N). These results illustrate that reduced fruit size may be caused by the decreased content of cytokinin and auxin.

Subsequently, DEGs involved in auxin and cytokinin pathways were isolated from fruit RNA-seq data (Figure 7A,B). The Dual-LUC assay identification showed that SlMADS48 could bind to the promoter of *SlIAA29* and *SlcycD6;1* via the CArG-box motif, further influencing their transcription level. This finding aligned with the qRT-PCR results showing the significantly downregulated expression of *SlcycD6;1* and *SlIAA29* in OE*-SlMADS48* lines compared with the WT (Figure 7E). Previous research found that the *fs8.1* mutant exhibits increased fruit size compared with the WT, together with the increased expression level of *SlIAA29* and other genes involved in the auxin pathway, illustrating the possible role of *SlIAA29* in fruit size determination [71]. In addition, the homologous gene of *SlIAA29* in *Arabidopsis* is *AtIAA29*, which has been found to function in cell division [72]. *SlcycD6;1* is a member of the cyclin gene family in tomato, and it has been found that D-type cyclins play a pivotal role in the transition from G1-S phase, further promoting cell division and growth [73,74]. Thus, SlMADS48 directly targets *SlcycD6;1* and *SlIAA29*, downstream genes that function in cell division, and inhibits their transcription, indicating its role in regulating cell division and determining fruit size.

### 3.4. Application in Future Breeding

In the present research, we found that the overexpression of *SlMADS48* generated elongated sepals compared with the wild type. In a previous study, the silencing of *SlMBP21* mediated by RNAi led to elongated sepals accompanied by the enhanced transcription of genes involved in photosynthesis, the accumulation of chlorophyll, and the activity of Rubisco, a key photosynthetic enzyme; these results demonstrate that elongation promotes photosynthesis, further improving the quality of the fruit [5]. This gene is expected to become a new target gene for improving tomato fruit quality.

In addition, in the present research, the overexpression of *SlMADS48* led to altered inflorescence together with an increased number of flowers. Although the *SlMADS48*-OE lines exhibited reduced fruit size, how to balance the fruit number and fruit weight would have effect on the final yield. In future research, we will focus on the yield of *SlMADS48*-OE lines. Moreover, we will combine traditional breeding and modern breeding methods, and create high-yield strains based on the biological functions of *SlMADS48*.

## 4. Materials and Methods

### 4.1. Plant Materials and Growth Conditions

In the current study, *Solanum lycopersicum* Mill. cv. Ailsa Craig served as the wild type (WT). All of the seedlings, including WT and transgenic-line plants, were cultivated in a standard greenhouse under the following conditions: a 16 h light/8 h dark cycle, 27 °C/18 °C day/night temperature, 80% relative humidity, and a photosynthetic photon flux density (PPFD) of 250 μmol m^−2^ s^−1^. Vegetative organs including the root, stem, and leaves (young, mature, senescent) and reproductive tissues covering flowers, sepals, and fruits at five stages (IMG [immature green], MG [mature green], B [breaker], B+4 [4 days after breaker], B+7 [7 days after breaker]) were gathered from WT tomato for a tissue-specific expression pattern analysis of *SlMADS48*. The four-whorl floral organs (sepals, petals, stamens, pistils) in the WT were also harvested. Two generations of plants were used to perform the function study: first-generation plants were obtained from tissue culture and second-generation plants were grown in soil. All samples involved in the study were rapidly frozen in liquid nitrogen and stored at −80 °C until use.

### 4.2. Construction of Expression Vector and Tomato Transformation

To generate the OE*-SlMADS48* vector, a 680 bp fragment covering the coding sequence (CDS) was amplified using *SlMADS48*-F and *SlMADS48*-R primers (Table S1). The amplified products were cloned in the plant binary expression vector *pBI121* under the control of the 35S promoter. The completed binary plasmid, verified by sequencing, was transformed into WT tomato using *Agrobacterium tumefaciens* strain LBA4404 [75]. Transgenic plants were rooted on Murashige and Skoog culture medium containing kanamycin (50 mg L^−1^) for selecting the positive transgenic line selection, and were verified by genomic PCR (polymerase chain reaction) using the kanamycin resistance gene *NPTII-F*/*R* primers (Table S1). The positive OE*-SlMADS48* transgenic lines were maintained for further studies.

For the *slmads48* mutant, the genome DNA sequence of *SlMADS48* was downloaded from the SGN Database (https://solgenomics.net/, accessed on 14 March 2025). Using CRISPR-P 2.0 (http://crispr.hzau.edu.cn/CRISPR2/, accessed on 14 March 2025), a guide RNA was designed such that it targeted the 5′-proximal coding regions to optimize editing efficiency. The guide RNA was subsequently inserted into pKSE401-CRISPR 9 and introduced into WT tomato by *Agrobacterium tumefaciens* strain LBA4404-mediated transformation. Following kanamycin selection and NPTII-F/R primer PCR, genomic DNA was extracted from positive transgenic lines. PCR was performed using primers spanning the protospacer-adjacent motif site and its flanking 300 bp regions. The mutation status was confirmed by the sequencing analysis of PCR products.

### 4.3. Extraction of Total RNA and Quantitative Real-Time PCR (qRT-PCR) Assay

Total RNA from the tested samples was extracted using the RNAiso Plus reagent kit (Takara, Tokyo, Japan) according to the manufacturer’s instructions. The cDNA was synthesized using M-MLV reverse transcriptase (Promega, Madison, WI, USA) and Oligo d(T)_20_ primer. The qRT-PCR assays were performed using the SYBR Premix Ex Taq II kit (Takara, Kyoto, Japan) in the CFX Connect Real-Time System (Bio-Rad, Hercules, CA, USA). All of the qRT-PCR reactions were performed in a 10 μL total sample volume, comprising 5 μL GoTaq qPCR Master Mix (Promega), 0.5 μL mixture primer, 1.0 μL cDNA, and 3.5 μL nuclease-free water. The PCR protocol consisted of 95 °C for 3 min, followed by 41 cycles of 95 °C for 15 s and 60 °C for 40 s, and then 95 °C for 1 min. *SlCAC* (*Solyc08g006960*), a tomato housekeeping gene with stable expression across organs, served as an internal control [76]. All of the gene-specific primers of related genes used for qRT-PCR analysis are listed in Table S1.

### 4.4. Histological Analysis

In the present study, the samples of sepals, whole flowers at −4DPA, and fruits at 25DPA were collected and fixed with FAA (formalin aceto alcohol, 70%, prepared with 5% formalin, 5% glacial acetic acid, and 90% 70% ethanol by volume). After incubation at 4 °C overnight and dehydration through an ethanol series, the samples were embedded in paraffin. Paraffin sections were prepared according to a previous report [5], and observations and photographs were taken using a microscope (OLYMPUS IX71; Olympus, Tokyo, Japan).

### 4.5. Determination of Endogenous Hormones in Plants

Fresh plant samples were harvested, immediately frozen in liquid nitrogen, ground into powder (30 Hz, 1 min), and stored at −80 °C until analysis. For each sample, 50 mg of plant sample was weighed into a 2 mL plastic microtube, frozen in liquid nitrogen, and dissolved in 1 mL methanol/water/formic acid (15:4:1, *v*/*v*/*v*). An internal standard was added as 10 μL of mixed solution (100 ng/mL) for quantification. The mixture was vortexed for 10 min, followed by centrifugation for 5 min (12,000 rpm, 4 °C). The supernatant was transferred to clean plastic microtubes, evaporated to dryness, dissolved in 100 μL 80% methanol (*v*/*v*), and filtered through a 0.22 μm membrane filter for further analyses. The sample extracts were analyzed using an UPLC-ESI-MS/MS (ultra-high-performance liquid chromatography–electrospray ionization–tandem mass spectrometry) system (UPLC, ExionLC™ AD; MS, QTRAP^®^ 6500+, Framingham, MA, USA). The analytical conditions were as follows: LC, column, Waters Acquity UPLC HSS T3 C18 (100 mm × 2.1 mm i.d.,1.8 µm); solvent system, water with 0.04% acetic acid (A), acetonitrile with 0.04% acetic acid (B); gradient program, 0 min A/B 95:5 (*v*/*v*), 1.0 min A/B 95:5 (*v*/*v*), 8.0 min 5:95 (*v*/*v*), 9.0 min 5:95 (*v*/*v*), 9.1 min 95:5 (*v*/*v*), 12.0 min 95:5 (*v*/*v*); flow rate, 0.35 mL/min; temperature, 40 °C; and injection volume, 2 μL [77,78,79].

### 4.6. RNA-Seq Analysis

In the present study, the samples from two independent wild type lines (WT-1 and WT-2) and two independent transgenic lines (OE-6 and OE-9) exhibiting high transcription levels of SIMADS48 were collected for RNA-seq analyses. Total RNA served as the input for the RNA sample preparation. Specifically, mRNA was isolated from total RNA using poly-T oligo-conjugated magnetic beads. Fragmentation was performed with divalent cations at an elevated temperature in First-Strand Synthesis Reaction Buffer (5X). First-strand cDNA synthesis utilized random hexamer primers and M-MuLV Reverse Transcriptase (RNase H^−^), followed by second-strand cDNA synthesis employing DNA Polymerase I and RNase H. Exonuclease and polymerase activities converted overhangs to blunt ends. Following the adenylation of 3′-ends of DNA fragments, adaptors with hairpin loop structures were ligated to enable hybridization. The AMPure XP system (Agencourt Bioscience Corp., Beverly, MA, USA) facilitated the selective purification of 370–420-bp cDNA fragments. Subsequently, PCR amplification employed Phusion High-Fidelity DNA Polymerase (Thermo Fisher Scientific, Waltham, MA, USA), universal PCR primers, and Index (X) Primer (New England Biolabs, Ipswich, MA, USA). The AMPure XP system was used to purify PCR products, and the Agilent Bioanalyzer 2100 system was used to assess library quality. Differential expression analysis was performed using the DESeq2 R package (v1.20.0), with genes exhibiting an adjusted *p*-value ≤ 0.05 and |log2(FoldChange)| ≥ 1 found by DESeq2 being assigned as differentially expressed genes (DEGs).

### 4.7. Yeast Two-Hybrid (Y2H) Assay

The Y2H experiment was performed as described previously [80]. The full-length coding sequence of *SlMADS48* was cloned into pGBKT7 to generate BD-SlMADS48, while the CDS of other test genes was inserted into the pGADT7 vector, respectively. Then, the constructed pGADT7 and pGBKT7 combination were transformed into Y2H Gold yeast strain using the lithium acetate method, with positive clones screened from double dropout (SD [solidified dextrose]/-Leu/-Trp DDO) medium and identified by PCR. Subsequently, the transformants, which were cultured in liquid-culture SD/Leu/Trp medium at 30 °C reaching OD_600_ = 0.5 (mid-log phase), were diluted (1, 1/10, and 1/100). Subsequently, 5 μL aliquots were then patched on SD/-Ade/-His/-Leu/-Trp plates containing 5-bromo-4-chloro-3-indolyl-α-D-galactopyranoside (X-α-Gal) for 3 days. Information about the primers used for the Y2H is given in Table S5.

### 4.8. Bimolecular Fluorescent Complementation (BiFC) Assay

A BiFC assay was performed to validate the Y2H interaction results [80]. The analysis used the expression vector pFGC-GFP and confocal laser scanning microscopy (CLSM; Olympus, Hachioji, Japan). The full-length CDS of *SlMADS48* without the stop codon was inserted into pFGC-nGFP to generate the fusion protein SlMADS48-GFPN, while the CDS of other identified genes without the stop codon was inserted into pFGC-cGFP. Following sequence confirmation, the constructed vector plasmids were transformed into *A. tumefaciens* strain GV3101, and *Nicotiana benthamiana* infiltration was performed using a modified method [81], with HY5-RFP serving as the nucleus-located marker. The results were observed by CLSM after 3 days. Table S6 contains the primers used for the BiFC assay.

### 4.9. Dual-LUC Assay

In this part, the CDS of *SlMADS48* was cloned into the pGreenII 62-SK vector as an effector, while the promoter fragment containing the CArG-box motif was inserted into the pGreenII 0800-GUS vector as a reporter, respectively. The Firefly luciferase (LUC) and Renilla luciferase (REN) activities were measured according to previous protocols [82]. Information about the primers used for the Dual-LUC assay are given in Table S7.

### 4.10. Electrophoretic Mobility Shift Assay (EMSA)

In this study, the CDS of *SlMADS48* was cloned into the pGEX4T-1 vector, generating a pGEX4T-1*-SlMADS48* fusion expression vector that was transformed into DE3 cells. The procedure utilized 0.2 mM isopropyl β-d-1-thiogalactopyranoside (IPTG) at 16 °C, 150 rpm, for 8 h. After sonication and protein purification, we obtained the purified GST-SlMADS48 fused protein. Probes were synthesized by Sangon Biotech (Shanghai, China) tagged with 5′-biotin, as shown in Table S8. The GST-MADS48 fusion protein was incubated with the probe and then subjected to PAGE (polyacrylamide gel electrophoresis). Subsequently, Western blotting was performed using chemiluminescent nucleic acid detection (Thermo Fisher Scientific, Waltham, MA, USA).

### 4.11. Chromatin Immunoprecipitation–Quantitative PCR (ChIP-qPCR) Assay

In the present study, the CDS of *SlMADS48* was cloned into a pCAMBIA2300-eGFP vector and the completed vector was transferred to the WT using the *Agrobacterium*-mediated leaf disc transformation method. After 1 month, 2 g of callus tissue was ground into powder in liquid nitrogen, followed by the addition of 36 mL ChIP extraction buffer 1, 1 mL of 37% formaldehyde, and 370 μL of 100 mM PMSF (phenylmethanesulfonyl fluoride). The mixture was crosslinked on a shaker for 10 min. Crosslinking was terminated by adding 2.5 mL of 2 M glycine. The sample was filtered through two layers of Miracloth (Calbiochem; Merck KGaA, Darmstadt, Germany) into a fresh 50 mL centrifuge tube, centrifuged, and the supernatant discarded. The pellet was resuspended in 600 μL of ChIP extraction buffer 2, followed by the gradual addition of 600 μL of ChIP extraction buffer 3. After centrifugation, the supernatant was discarded. The pellet was subsequently resuspended in 600 μL of nuclear lysis buffer and 5.4 mL of ChIP dilution buffer. Sonication was performed at 90–120 W, alternating 5 s of sonication with 5 s of rest, for 10–15 min. Protein A+G magnetic beads were prepared and added to the lysate. After washing with high-salt elution buffer and TE buffer, the beads were resuspended, and the supernatant was discarded. ChIP elution buffer was then added, and the mixture was incubated in a 65 °C water bath for 10 min. Finally, 9 μL of 10% SDS (sodium dodecyl sulfate) was added to the sample, and the mixture was subjected to reverse crosslinking in a 65 °C water bath for 6–8 h. Specific primers were designed based on the CArG-box motif in the promoter region of the target gene, and subsequent experiments were conducted using the previously described quantitative PCR method. The primers for the ChIP-qPCR assay were given in Supplementary Table S8.

### 4.12. Statistical Analysis and Heatmap Drawing

For all experiments in this study, measurements from three independent biological replicates were used for statistical analysis, and significant differences among the means were determined using the *Student’s t*-test in Prism (v 8.0.2; GraphPad Software, Inc., San Diego, CA, USA). Heatmap visualization was performed using TBtools (v1.120; South China Agricultural University, Guangzhou, Guangdong, China).

## 5. Conclusions

Collectively, the present study explored the function of an uncharacterized MADS-box TF gene, *SlMADS48*. The overexpression of *SlMADS48* generated more elongated sepals compared with the WT, together with an increased content of gibberellin. Moreover, the protein–protein interaction between SlMADS48 and JOINTLESS, MBP21, MC, and FYFL, which are involved in sepal development, were identified. Moreover, the inflorescence structure was altered with increased numbers of branches and flowers. Subsequently, the interaction between SlMADS48 and TM29, FUL1, FUL2, and MBP20 was identified, and SlMADS48 was found to possess the ability to bind to the promoter and inhibit the transcription of *SlTM3*. In addition, the overexpression of *SlMADS48* led to a decrease in fruit size compared with the WT, together with a decrease in the accumulation of IAA and cytokinin. Furthermore, it was found that SlMADS48 directly targeted and inhibited the transcription of *SlIAA29* and S*lcycD6;1*. These findings establish a foundation for exploring tomato MADS-box family genes and provide potential reference genes for tomato breeding.

## Data Availability

The original contributions presented in this study are included in the article/Supplementary Materials. Further inquiries can be directed to the corresponding authors.

## Supplementary Materials

The following supporting information can be downloaded at: https://www.mdpi.com/article/10.3390/plants14213259/s1, Figure S1: The bioinformatic analyzes of SlMADS48 and the transcript level of SlMADS48 in young leaves of positive transgenic lines; Figure S2: The detection of autoactivation activity and the RNA-seq analyzes of sepals between WT and SlMADS48-overexpressed lines; Figure S3: Y2H assay illustrated that SlMADS48 didn’t interacted with FA, WUS, and AHL15, which participated in the inflorescence development; Figure S4: The Dual-LUC assay schematic diagram; Figure S5: The statistical data on agronomic traits of fruits from SlMADS48-overexpressed lines and mutant lines at both 25 DPA and B+4 stages, and the expression patterns of genes involved in auxin, cell division, and cell wall development in 25 DPA fruits of the SlMADS48-overexpressed lines; Figure S6: The RNA-seq information of 25 DPA between SlMADS48-overexpressed lines and WT; Figure S7: Dual assay of SlMADS48 to SlcycD6;1 and SlIAA29; Table S1: The primer for qRT-PCR assay; Table S2: *cis*-acting elements in 3000 bp promoter region before ATG; Table S3: RNA-seq data summary of sepals; Table S4: RNA-seq data summary of fruits; Table S5: The primer sequence for yeast two hybrid experiment; Table S6: The primer sequence for BiFC experiment; Table S7: Primers for Dual-LUC assay; Table S8: Primers for EMSA; Table S9: Primers for ChIP-qPCR assay.
