# Supplementary material for: Overexpression of SlMADS48 Alters the Structure of Inflorescence and the Sizes of Sepal and Fruit in Tomato
Source: Plants (Basel). 2025 Oct 24;14(21):3259. doi: 10.3390/plants14213259 (PMC12608182; doi:10.3390/plants14213259)
Supplement: Supplementary file 1 [file plants-14-03259-s001.zip › plants-3914580-supplementary.pdf]

Overexpression of *SlMADS48* Alters the Structure of Inflorescence and the Sizes of Sepal and Fruit in Tomato

Pengyu Guo [1], Xin Cheng [1], Chuanji Xing [2], Zihan Gao [1], Jing Xue [2], Xiuhai Zhang [2], Guoping Chen [1], Xuqing Chen [2,*] and Zongli Hu [1,*]

[1] Laboratory of Molecular Biology of Tomato, College of Bioengineering, Chongqing University, Chongqing 400044, China; guopengyucqu@163.com (P.G.); chengxin599@163.com (X.C.); gzhfilm@163.com (Z.G.); chenguoping@cqu.edu.cn (G.C.)

[2] Institute of Grassland, Flowers and Ecology, Beijing Academy of Agriculture and Forestry Sciences, Beijing 100097, China; 18704404680@163.com (C.X.); xuejing@baafs.net.cn (J.X.); zhangxiuhai@baafs.net.cn (X.Z.)

* Correspondence: chenxuqing@baafs.net.cn (X.C.); huzongli@cqu.edu.cn (Z.H.); Tel.: +86-010-51503829 (X.C.); +86-2365102507 (Z.H.)

Figure S1

A

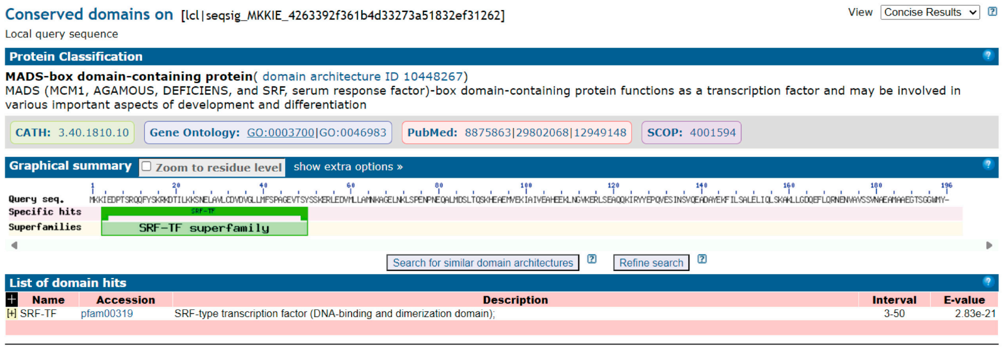

B

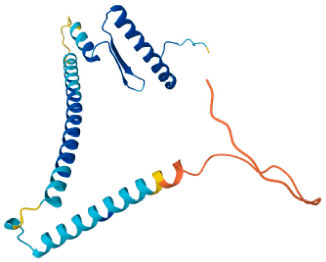

C

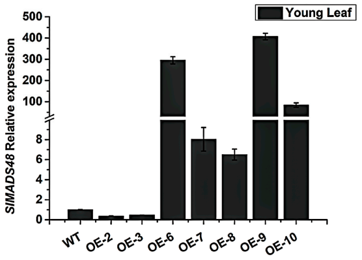

Figure S1. The bioinformatic analyzes of SlMADS48 and the transcript level of *SlMADS48* in young leaves of positive transgenic lines.

(A) The conserved domain research based on the protein sequence of SlMADS48.

(B) The three-dimensional structure of SlMADS48 based on protein sequence using online tool SWISS Model (https://swissmodel.expasy.org/interactive).

(C) The transcript level of *SlMADS48* in leaves which were transgenic overexpressed lines. Data were the meant ± SE.

Figure S2

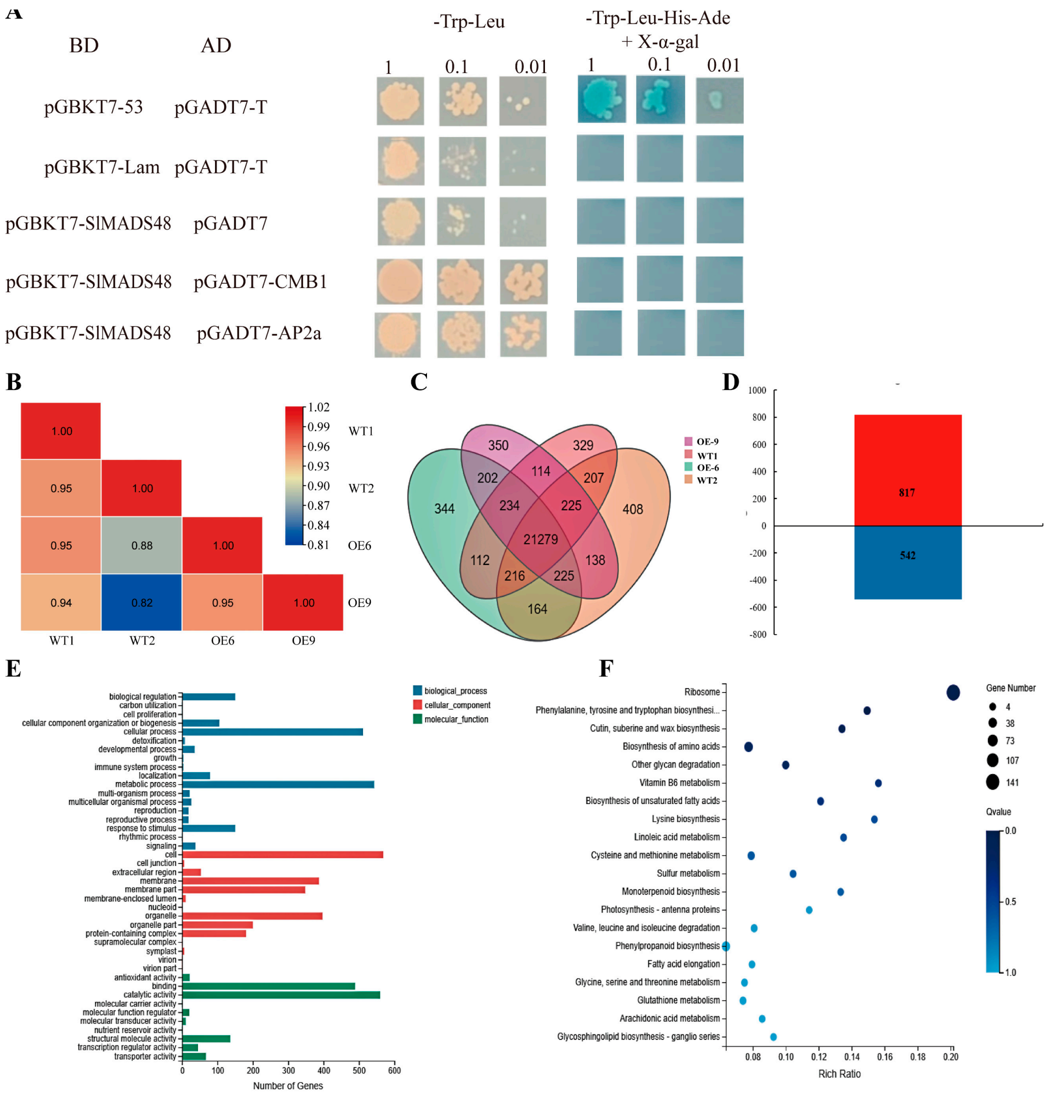

Figure S2. The detection of autoactivation activity and the RNA-seq analyzes of sepals between WT and *SlMADS48*-overexpressed lines.

(A) The yeast two-hybrid assay demonstrated that this protein lacked autoactivation activity and SlMADS48 didn't interact with CMB1 and AP2a. The leftmost column represents the bacterial culture at OD_{600}=1, with subsequent columns containing 10-fold and 100-fold serial dilutions for spot assay. Photographs were taken after 5 days of inverted incubation at 30°C.

(B) The Pearson correlation coefficient was calculated to evaluate the correlation between the four samples. (C) A Venn diagram illustrates the overlap and unique expressed genes identified in the samples. (D) The number of differentially expressed genes (DEGs) between wild type and *SlMDAS48*-overexpresssed lines, with 817 up-regulated and 852 down-regulated DEGs. (E) Gene Ontology analyses were performed on the DEGs to determine their functional categories. (F) The Kyoto Encyclopedia of Genes and Genomes (KEGG) enrichment analysis was conducted to identify enriched pathways among the major up-regulated and down-regulated DEGs.

Figure S3

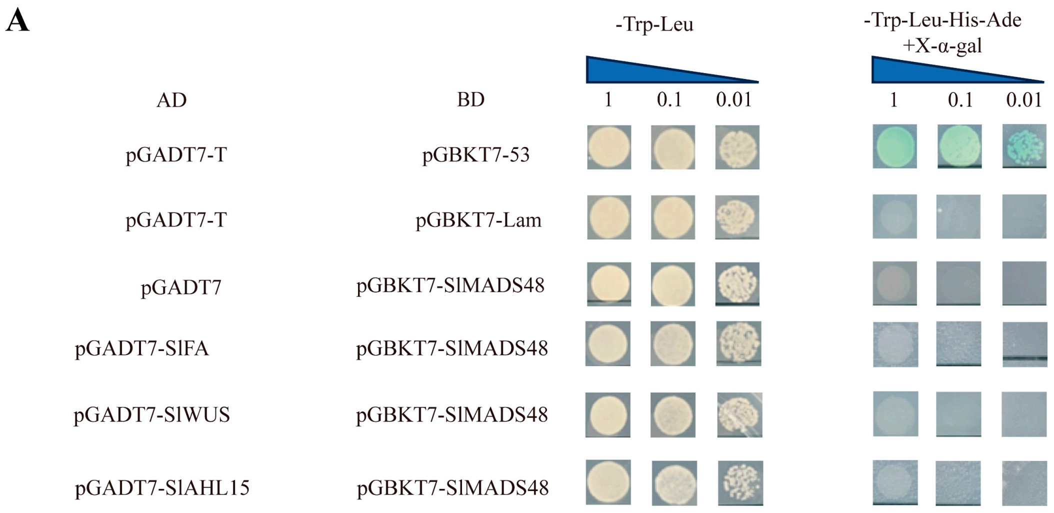

Figure S3. Y2H assay illustrated that SlMADS48 didn't interacted with FA, WUS, and AHL15, which participated in the inflorescence development.

(A) The protein interaction didn't be observed between SlMADS48 and FA, WUS, AHL15 which were involved in the inflorescence development. SlMADS48 transcription factor protein lacked transcriptional activation activity. The leftmost column represents the bacterial culture at OD_{600}=1, with subsequent columns containing 10-fold and 100-fold serial dilutions for spot assay. Photographs were taken after 5 days of inverted incubation at 30°C.

Figure S4

A

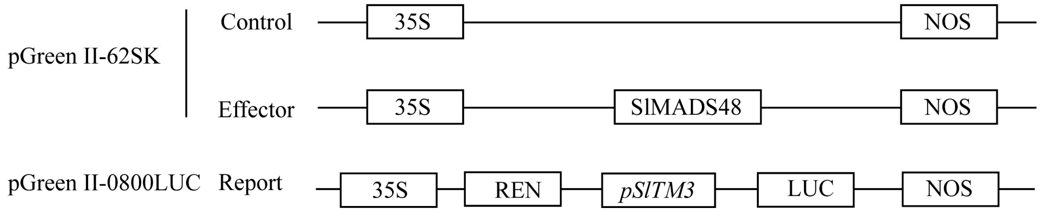

Figure S4. The Dual-LUC assay schematic diagram.

(A) Schematic diagram of the Dual-LUC experiment of SlMADS48 on *SlTM3*.

Figure S5

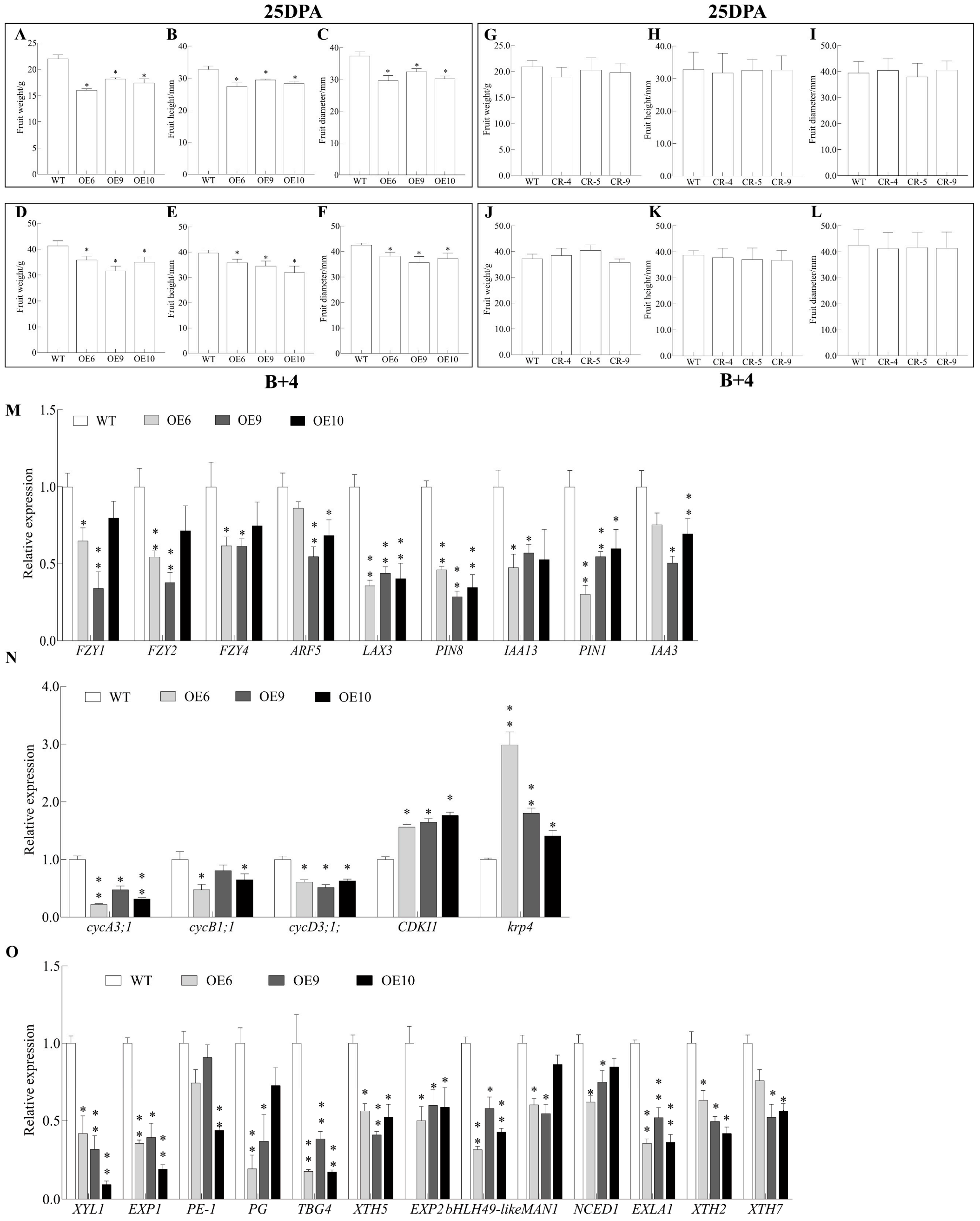

Figure S5. The statistical data on agronomic traits of fruits from SlMADS48-overexpressed lines and mutant lines at both 25 DPA and B+4 stages, and the expression patterns of genes involved in auxin, cell division, and cell wall development in 25 DPA fruits of the SlMADS48-overexpressed lines.

(A-F) The comparison about weight, height, and diameter of fruit at 25DPA (A-C) and B+4 stage (E-F) between WT and OE lines.

(G-L) The comparison about weight, height, and diameter of fruit at 25DPA (A-C) and B+4 stage (E-F) of between WT and CR lines.

(M-O) The qRT-PCR results of genes between WT and OE lines involved in auxin, cell division and cell development.

Data were the meant ± SE. * indicated significant difference analysis, *Student's t*-test $P<0.05$, ** $P<0.01$.

Figure S6

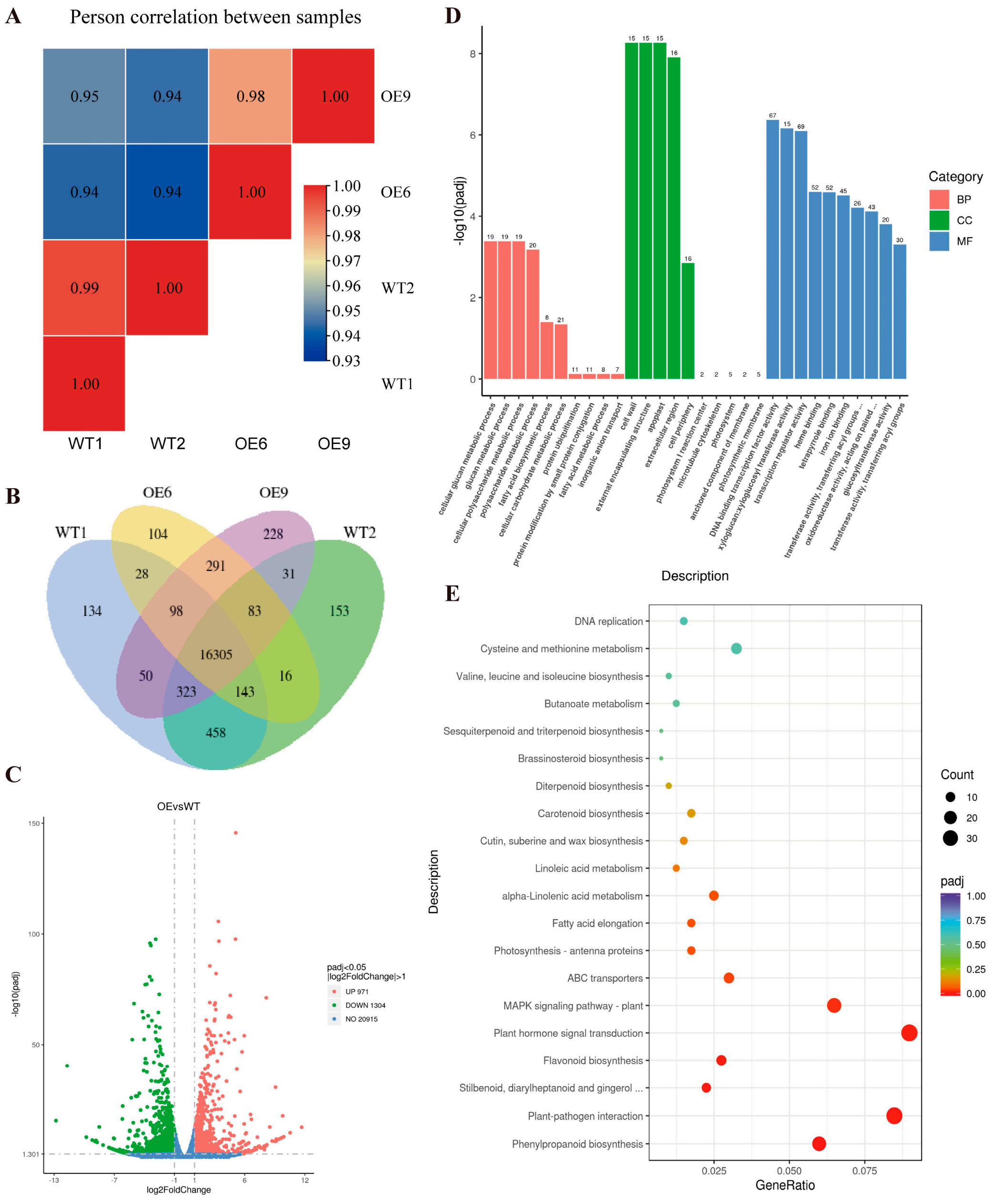

Figure S6. The RNA-seq information of 25 DPA between *SlMADS48*-overexpressed lines and WT.

(A) The Pearson correlation coefficient of four samples for testing. (B) A Venn diagram illustrated the overlap and unique expressed genes identified in the samples. (C) The volcano plot showed the number of differentially expressed genes (DEGs) between WT

and OE lines, with 817 up-regulated and 852 down-regulated DEGs. (D) Gene Ontology analyses were performed on the DEGs to determine their functional categories. (E) The Kyoto Encyclopedia of Genes and Genomes (KEGG) enrichment analysis was conducted to identify enriched pathways among the major up-regulated and down-regulated DEGs.

Figure S7

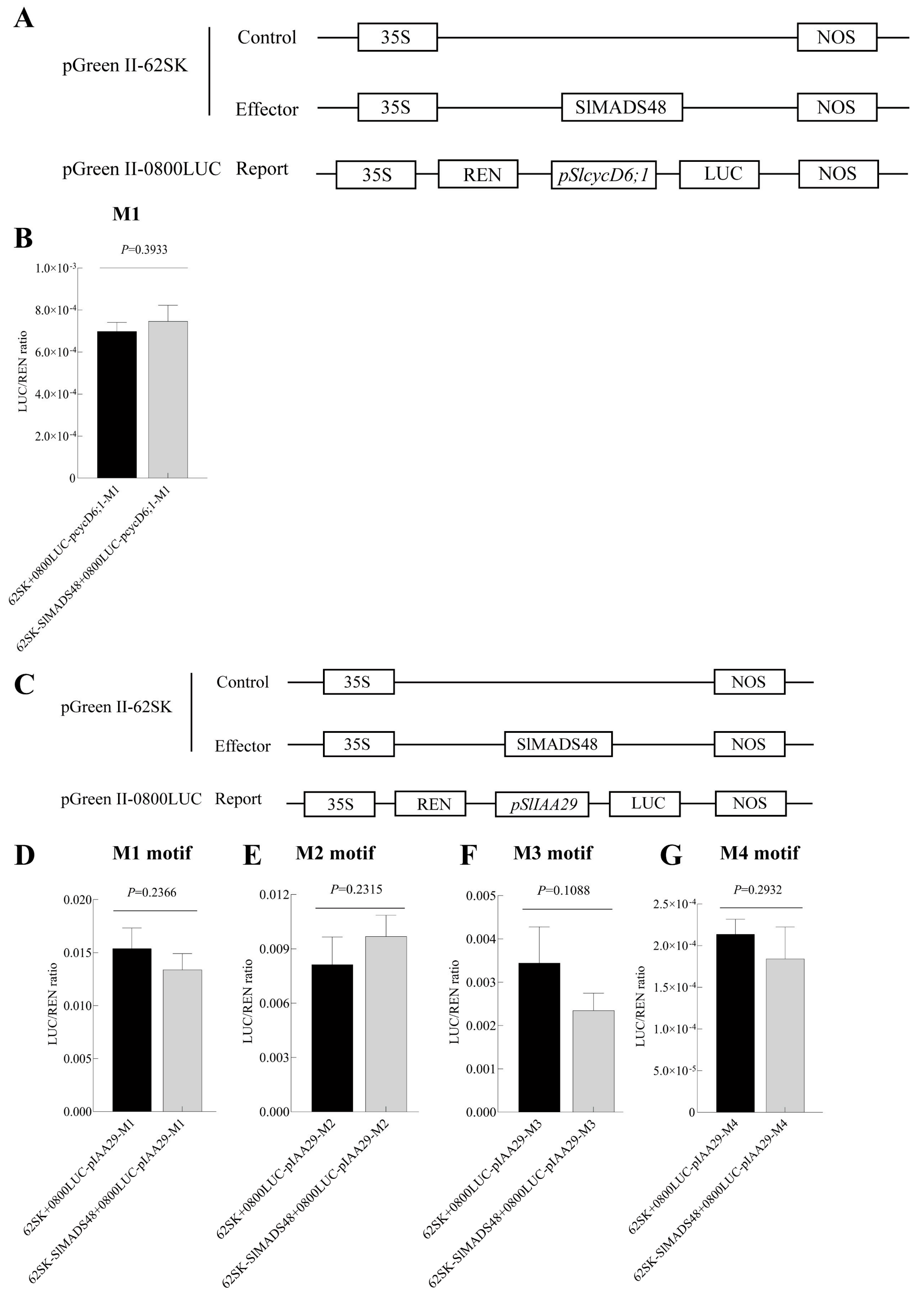

Figure S7. Dual assay of SlMADS48 to *SlcycD6;1* and *SlIAA29*.

(A) Schematic diagram of the Dual-LUC experiment of SlMADS48 on *SlcycD6;1*.

(B) The Dual-LUC assay results of SlMASD48 on M1 motif in promoter of *SlcycD6;1*.

(C) Schematic diagram of the Dual-LUC experiment of SlMADS48 on *SlIAA29*.

(D-G) The Dual-LUC assay results of SlMASD48 on M1 (D), M2 (E), M3 (F), and M4 (G).

Table S1. The primer for qRT-PCR assay

Primer name	Sequence (5'-3')
SlMADS48-F	GCTCTAGAGACATTGAGTTCTGCTTGGTTG
SlMADS48-R	CGAGCTCTGATCCGGCCTGTTTCCT
NPTII-F	CTCAGAAGAACTCGTCAAGAAGG
NPTII-R	GACTGGGCACAACAGACAATC
Q- *SlMADS48*-F	GTTGGCTGTTTTATGTGATGTGG
Q- *SlMADS48*-R	AGAGAGTCCATCAAAGCCTGTTC
Q-*CycA3;1*-F	CTAAGAAAAGAGCAGCAGAAGCA
Q-*CycA3;1*-R	GATTCCTTATCTTTTTCAGCAACAG
Q-*CycB1;1*-F	GTATCTCGCCCCGTAACAAG
Q-*CycB1;1*-R	TCTCCTCAGGTTTTGGCTTT
Q-*CycD3;1*-F	CTGCCAAAGCCTCAAGCG
Q-*CycD3;1*-R	CAGTGGAGCTAGTGTCATTCGC
Q-*Krp4*-F	CACAAGGAAGAGGAAGAAGCG
Q-*Krp4*-R	CCAAAACCAGATGCTGAAACG
Q-*GA20ox1*-F	TTCTCAAATTGGCTTCATGATCAA
Q-*GA20ox1*-R	TTCCCCCTAATTCCCATAACAT
Q-*GA20ox2*-F	TAAGAAGGATAAGGTGGTGAGGC
Q-*GA20ox2*-R	CCGTAGTTTTCTGTTGAAGCCA
Q-*GA3ox1*-F	ATAGGCACCCACCCTTGTATA
Q-*GA3ox1*-R	GGATGAAAGTGCCTTGTCAAAAT
Q-*GID1*-F	TGCTAATGAATCTAAGAGGGTGG
Q-*GID1*-R	GTGCCATCAGGACGACGAA
Q-*CPS*-F	AGGTCTTGTTTTGGCTCCCC
Q-*CPS*-R	CAAGTAGTGATGGATGTCTCTGCC
Q-*KAO*-F	TGGACTTACACCAAAGGTAGGAA
Q-*KAO*-R	AAATACATCACTGGACAAGACGG
Q-GA2ox2-F	ATTAAGATCCAATAACACTTCG
Q-GA2ox2-R	TCTTGATTTCACACTATTTGC
Q-GA2ox4-F	ATTCTTCTCCTCTCCCCTCTCTGA
Q-GA2ox4-R	GACACATAATCATTCACCGCAGC
Q-GID1b-2-F	TGCTAATGAATCTAAGAGGGTGG
Q-GID1b-2-R	GTGCCATCAGGACGACGAA
Q-AN-F	AGGGACTAATAACTGGTGCAAGAT
Q-AN-R	TAAAACAACCTTACCATTCCCTTC
Q-SP-F	ACATTCCAGGCACTACAGATTG
Q-SP-R	GCAAAAATACAAACCTGTGGAT
Q-TMF-F	ACAACCCTAAGCCGATACGAGA
Q-TMF-R	GTGAGAGAGAGTGGTGGTCTATGG
Q-SFT-F	TTTTGGGCAAGAAATAGTGAGC
Q-SFT-R	ATACACTGTTTGCCGACCTAAT
Q-S-F	TAACGAGTTCCTGTCTAACGAGC
Q-S-R	GAATCCTGGAAGCAAAACCC

Q-FUL1-F	AAAATCAGTGGGAAATCAACTCATC
Q-FUL1-R	CCTTGCTGCTGTGAAGAACTACC
Q-WUS-F	TTTGGCTGCTCTTGAACTTACTCT
Q-WUS-R	ACATTACCATAAGGTCCAAATAGC
Q-MBP10-F	AAAGAAATATAAGGCATTTTATGGGA
Q-MBP10-R	CACTCGCTTTAGAGCTGTATCGAG
Q-AP2b-F	CCAATATATGAGAGCTGGTGACTCC
Q-AP2b-R	TGCTGAGGGAATCCTGATGATG
Q-SOC1-F	TCGAAAAGCTGGCTTAGTCAAGA
Q-SOC1-R	TCTTAGTCCGCCCATCCTGT
Q-ALH15-F	GAACGGGTTCAGTATTTCATTAGC
Q-ALH15-R	AACGTCGTTGCAATTAAATAAACC
Q-STM3-F	ACTATTAGGGCTCGAAAGATGCAA
Q-STM3-R	TCCAAACTTTAGTTCCCTCAGCAT
Q-BL-F	GGAAGATTTGATAGTTGTGTTG
Q-BL-R	CAAAAATAGAGCTACACAAAACC
Q-FUL2-F	CCGTGGGAGCAACAGAGTCAT
Q-FUL2-R	GGAGGCATCACAGAAGCACTG
Q-TM29-F	TCAAAGACACCTTCTTGGAGATGAG
Q-TM29-R	GGACCTAATGTGTTTGAGGGAAGTA
Q-TAP3-F	TATAAGTCCCTCAATCACGACCA
Q-TAP3-R	GATCATTTAGGCTTTCTCCCATC
Q-TM6-F	CTACAACCATTGCACCCCAAT
Q-TM6-R	CAGGAGAGACGTAGATCACGAGAA
Q-AGL6-F	GCTTCGTAGAAAGGAGCGTCAT
Q-AGL6-R	GATTTGATTGAGAATGGTGGACATC
Q-SEP3-F	CTTTGTGATGCTGAGGTTGCTC
Q-SEP3-R	TTTCCAGTGCTTCTCGTGTTG
Q-MBP20-F	GAAGCTAAAAGAAAATGAGAAGACACA
Q-MBP20-R	GTAAGGTTAGGAAGTTGGTGGTGAG
Q-XYL1-F	TGATCGGCAATTATGAAGGTATTC
Q-XYL1-R	CAGCACATCCTGGCTTGTAAAT
Q-EXP1-F	CCCTCCTCGCCCTCACTTT
Q-EXP1-R	TCTGATTCCTCCTTGCTTTCG
Q-PE-1-F	GCTTGCGTCTTTGACAACTCAGG
Q-PE-1-R	GTGCCACCACTGCATTCGCTAT
Q-PG-F	ATACAACAGTTTTCAGCAGTTCAAGT
Q-PG-R	GGTTTTCCACTTTCCCCTACTAA
Q-TBG4-F	AAATGGTGAAGGCGTAGGTCG
Q-TBG4-R	AGGTTGTCCGCAGTTAGTCTGG
Q-*AP2a*-F	GAATGTACTGATAATGCAACGGACC
Q-*AP2a*-R	GCTGCTCGGAGTCTGAACCTTA
Q-*SlCMB1*-F	TGAGCGTCAACTGGATTCATCTT
Q-*SlCMB1*-R	CCCTCTGACTGAGCAGGTTGTT

Q-*SlFYFL*-F	GCAAGAGAAACAACGACGGTG
Q-*SlFYFL*-R	CCAGTTGGCTGTCAATGTCTTCTA
Q-*GOBLET*-F	CTGAACTTGACTGTATGTGGAGC
Q-*GOBLET*-R	GAACGTTTACAACAAAGTGACAAT
Q-*SlMBP21*-F	AACCTTTCTTTCAACCTCTCCG
Q-*SlMBP21*-R	TCCATTAGAGCATCCACCCTG
Q-*MC*-F	AAGTAGCAGAAGCAAGGAGGA
Q-*MC*-R	CAAGCGATTAGCAAAGAGTGA
Q-*JOINTLESS*-F	AAGGCAACAGGTGATGGAGATA
Q-*JOINTLESS*-R	GCCTGAGTAAGGTAGCCCCA
Q-*XTH6*-F	GTCCCCCTTCACCTGGCTAC
Q-*XTH6*-R	TCCCAAAATAACCGGAACGATA
Q-*CDKI1*-F	TCCGGTGGTGAGTTCTGATAAGTAT
Q-*CDKI1*-R	CAACTTCCCCACACTTCTCATCTA
Q-*SlCAC*-F	CCTCCGTTGTGATGTAACTGG
Q-*SlCAC*-R	ATTGGTGGAAAGTAACATCATCG
Q-XTH5-F	CCACCACCAGAGTGCGAGAT
Q-XTH5-R	TTTTCTTAGGATGACGATGTCCG
Q-EXP2-F	TGGCTTCACTTCCACTTGTTTT
Q-EXP2-R	CCATAGAAAGTGGCATGAGCAG
Q-bHLH49-like-F	TGGAGTCTCTGGTAATGAGTCTGATG
Q-bHLH49-like-R	TGGCTGCTGTGCTCCCTTC
Q-MAN1-F	ACACCGTCCTCCTGAGATTGG
Q-MAN1-R	GAGCCTCTGCTTTCCACTTTAATC
Q-NCED1-F	CCCGATTTGGTATTCTGGATAAGTA
Q-NCED1-R	GAGACGGATTTCGGATAAAACACT
Q-EXLA1-F	CTGTTCCTAAACTTTACGATAATGGTG
Q-EXLA1-R	TGAACAAATTTTTGGGTCCGTAC
Q-XTH2-F	TGTTTCTTCGTAGTGGTGGCT
Q-XTH2-R	AAGAAGTTGCCCGTTTTCG
Q-XTH7-F	CGACCCGCCACTTTTTTACAG
Q-XTH7-R	AACGAGCTTGATCTTCATGCTAACA
Q-FZY1-F	CTGTAGACATAACGCCATTCCTCAT
Q-FZY1-R	AGGGCCATAGCTATTGAAAATGTTGA
Q-FZY2-F	AGGAATGGAGGTGTGTTTGG
Q-FZY2-R	GGGACGTGTCACCGAGTAA
Q-FZY4-F	TCGATTCTGTTCTTCTTGCTACT
Q-FZY4-R	CTGATAGTCCTCTTCTTGTGAAA
Q-ARF5-F	GAAGAGAAGAACAAGCCAGGGAGT
Q-ARF5-R	CAAGCATGCCACAACTCAGAACTA
Q-LAX3-F	TATTGGGCTTTTGGAGATGC
Q-LAX3-R	CAATGGGGTACATGCAAATC
Q-PIN1-F	GCTGCAGGCTGGTCTAGATT
Q-PIN1-R	AACAATGGCAACAAAGCACA

Q-PIN8-F	GGTGGGGGAAACTCTTTTCA
Q-PIN8-R	TTCCTTTGGGCTTCAGTTTG
Q-IAA3-F	GCCACCAGTTCGATCATACA
Q-IAA3-R	ATAAGGTGCTCCATCCATGC
Q-IAA13-F	TGTTTCTGGTACTAAAAGAGCTGCT
Q-IAA13-R	GCTGTTCATTCTGTATGCCCTTAT
Q-tXET-B2-F	CTACCTTACACGGTTCATACCAATG
Q-tXET-B2-R	CACCAAAAATATGATCCGTTGAG
Q-IAA29-F	TAGGAAGGAAGGTTGATCTAAGGCT
Q-IAA29-R	CATGGTACATCTCCAGCAAGCAT
Q-cycd3c3-F	GAATTGTCAAGGAGAAGGTGGAA
Q-cycd3c3-R	ATGACATATCCATAACCCCTGTTG
Q-CDC20.2-F	TTCAGAATTGGTCACTGTTGACG
Q-CDC20.2-R	ATAGTTCTCAGCAGCCGATTTG

Table S2. *cis*-acting elements in 3000 bp promoter region before ATG

***cis*-elements**	**sequence**	**numbers**	**functions**
ABRE	ACGTG	1	Response to abscisic acid
AuxRR-core	GGTCCAT	1	Response to auxin
G-box	CACGAC	2	Response to light
MBS	CAACTG	1	MYB binding site by drought induced
TATC-box	TATCCCA	3	Response to gibberellin
TCA-element	CCATCTTTTT	1	Response to salicylic acid
TGACG-motif	TGACG	3	Response to MeJA
circadian	CAAAGATATC	1	Circadian rhythm
ARE	AAACCA	1	Anaerobic induction
CAT-box	GCCACT	1	Meristem induction
GCN4_motif	TGAGTCA	1	Endosperm expression
HD-Zip 1	CAAT(A/T) ATTG	2	Palisade mesophyll cell differentiation

Table S3. RNA-seq data summary of sepals

Samples	Total Raw Reads (M)	Total Clean Reads (M)	Total Clean Bases (Gb)	Clean Reads Q20 (%)	Clean Reads Q30 (%)	Clean Reads Ratio (%)
WT1	45.22	43.67	6.55	93.84	87.06	96.57
WT2	45.18	43.81	6.57	93.60	86.59	96.97
OE-6	45.07	43.98	6.60	93.94	87.27	97.57
OE-9	44.90	43.37	6.51	93.90	87.14	96.60

Table S4. RNA-seq data summary of fruits

Samples	Total Raw Reads (M)	Total Clean Reads (M)	Total Clean Bases (Gb)	Clean Reads Q20 (%)	Clean Reads Q30 (%)
WT1	46.73	45.78	6.87	97.82	93.58
WT2	48.04	47.17	7.08	98.04	94.41
OE-6	46.82	46.29	6.94	98.19	94.41
OE-9	46.34	44.98	6.75	98.23	94.65

Table S5. The primer sequence for yeast two hybrid experiment.

Primer name	Sequence (5'-3')
Y2H-SlMADS48-F	CGGAATTC ATGAAGAAAATTGAGGATCCAACAT
Y2H-SlMADS48-R	TCCCCCGGG TCAATACATCCATCCCCCACTG
Y2H-CMB1-F	CCGGAATTC ATGGGAAGAGGTAAGGTAGAATTGA
Y2H-CMB1-R	CGCGGATCC TCAAAGCATCCATCCTGGTAAA
Y2H-MC-F	CCGGAATTC ATGGGAAGAGGAAAAGTTGAATTA
Y2H-MC-R	CGCGGATCC TCATAGATGTTTATTCATGTTGTAAAGTG
Y2H-JOINTLESS-F	CCGGAATTC ATGGCTAGAGAAAAAATTCAGATC
Y2H-JOINTLESS-R	CGCGGATCC TCAGCCTGAGTAAGGTAGCCC
Y2H-MBP21-F	GGGAATTCCATATGATGGGAAGAGGAAGAGTAGAACTA
Y2H-MBP21-R	CGCGGATCC TTAGAGCATCCACCCTGGA
Y2H-AP2a-F	CCGGAATTC ATGTGGAATTTAAATGATTCCCCT
Y2H-AP2a-R	CGCGGATCC TCAAGGTCTCATAAAATAATGATGGA
Y2H-FYFL-F	CCGGAATTC ATGGTGAGAGGAAAAGTAGAAATGAA
Y2H-FYFL-R	CGCGGATCC CTATAAGCAGCGCGTTTGAGG
Y2H-FUL1-F	gccatggaggccagtgaattcATGGGAAGAGGAAGAGTCCAGTT
Y2H-FUL1-R	cagctcgagctcgatggatccTTAATTATTAAGATGACGAAGCATCCA
Y2H-FUL2-F	gccatggaggccagtgaattcATGGGTAGAGGAAGAGTACAATTGAA
Y2H-FUL2-R	cagctcgagctcgatggatccTTAACCGTTGAGATGGCGAAG
Y2H-AGL19-F	gccatggaggccagtgaattcATGGTGAGAGGAAAAACTGAGTTGA
Y2H-AGL19-R	cagctcgagctcgatggatccTTAAAGTAATGTAGGTAGTGTGTTTAAATTAGG
Y2H-MBP20-F	gccatggaggccagtgaattcATGGGAAGAGGTAGGGTAGAGTTG
Y2H-MBP20-R	cagctcgagctcgatggatccTCATCCTTCGTTGCTGACGTG
Y2H-DEF-F	gccatggaggccagtgaattcATGGCTCGTGGTAAGATCCAGA
Y2H-DEF-R	cagctcgagctcgatggatccTCAACCTAGAGCAAAAGTAGTAATATCAGA
Y2H-TM29-F	gccatggaggccagtgaattcATGGGTAGAGGAAGAGTTGAGCTG
Y2H-TM29-R	cagctcgagctcgatggatccTCACAGCATCCAACCAGGTATCA
Y2H-FA-F	gccatggaggccagtgaattcATGGACCCAGATGCTTTCTCG
Y2H-FA-R	cagctcgagctcgatggatccTTAGAAATGTGGCAGGTGATCAGC
Y2H-WUS-F	gccatggaggccagtgaattcATGATGGAACATCAACACAACATAGA
Y2H-WUS-R	cagctcgagctcgatggatccTTAGGGGAAAGAGTTGAGAGTAAGTTC
Y2H-AHL15-F	gccatggaggccagtgaattcATGAAAGAAAAATATATAGAAGAAAGAAAAAA
Y2H-AHL15-R	cagctcgagctcgatggatccTTAGTAAGGCAGTAGTTGTCTAGGGG

Y2H-MBP10-F	gccatggaggccagtgaattcATGGGGCGGGGTAGGGTG
Y2H-MBP10-R	cagctcgagctcgatggatccTCATCCTTTGTTGTGGACATGG
Y2H-AP2d-F	gccatggaggccagtgaattcATGATGTTGGATCTCAATCTAAATGC
Y2H-AP2d-R	cagctcgagctcgatggatccTTATGGAGGTGGTAGTTGTGGCC

Table S6. The primer sequence for BiFC experiment.

Primer name	Sequence (5'-3')
BiFC-SlMADS48-F	CGAGCTC ATGAAGAAAATTGAGGATCCAACAT
BiFC-SlMADS48-R	TGCTCTAGA ATACATCCATCCCCCACTG
BiFC-MC-F	CGAGCTC ATGGGAAGAGGAAAAGTTGAATTA
BiFC-MC-R	TGCTCTAGA TAGATGTTTATTCATGTTGTAAAGTG
BiFC-JOINTLESS-F	CGAGCTC ATGGCTAGAGAAAAAATTCAGATC
BiFC-JOINTLESS-R	TGCTCTAGA GCCTGAGTAAGGTAGCCC
BiFC-MBP21-F	CGAGCTC ATGGGAAGAGGAAGAGTAGAACTA
BiFC-MBP21-R	TGCTCTAGA GAGCATCCACCCTGGA
BiFC-FYFL-F	CGAGCTC ATGGTGAGAGGAAAAGTAGAAATGAA
BiFC-FYFL-R	TGCTCTAGA TAAGCAGCGCGTTTGAGG
BiFC-FUL1-F	ctctcgagctttcgcgagctcATGGGAAGAGGAAGAGTCCAGTT
BiFC-FUL1-R	gatggatcttctagaggatccATTATTAAGATGACGAAGCATCCATT
BiFC-FUL2-F	ctctcgagctttcgcgagctcATGGGTAGAGGAAGAGTACAATTGAA
BiFC-FUL2-R	gatggatcttctagaggatccACCGTTGAGATGGCGAAGC
BiFC-AGL19-F	ctctcgagctttcgcgagctcATGGTGAGAGGAAAAACTGAGTTGA
BiFC-AGL19-R	gatggatcttctagaggatccAAGTAATGTAGGTAGTGTGTTTAAATTAGGAC
BiFC-MBP20-F	ctctcgagctttcgcgagctcATGGGAAGAGGTAGGGTAGAGTTG
BiFC-MBP20-R	gatggatcttctagaggatccTCCTTCGTTGCTGACGTGGC
BiFC-def-F	ctctcgagctttcgcgagctcATGGCTCGTGGTAAGATCCAGA
BiFC-def-R	gatggatcttctagaggatccACCTAGAGCAAAAGTAGTAATATCAGAGC
BiFC-TM29-F	ctatctctctttcgcgagctcATGGGTAGAGGAAGAGTTGAGCTG
BiFC-TM29-R	ggtggcgatggatcttctagaCAGCATCCAACCAGGTATCATACC
BiFC-MBP10-F	ctctcgagctttcgcgagctcATGGGGCGGGGTAGGGTG
BiFC-MBP10-R	gatggatcttctagaggatccTCCTTTGTTGTGGACATGGTGG
BiFC-AP2d-F	ctctcgagctttcgcgagctcATGATGTTGGATCTCAATCTAAATGC
BiFC-AP2d-R	ggtggcgatggatcttctagaTTATGGAGGTGGTAGTTGTGGCC

Table S7. Primers for Dual-LUC assay

ID	sequence
62SK-SlMADS48-F	tcccccgggctgcaggaattcATGAAGAAAATTGAGGATCCAACAT
62SK-SlMADS48-F	tcagcgtaccgaattggtaccTCAATACATCCATCCCCCACTG
pTM3-M1-F	ctatagggcgaattgggtaccACTAAAGACTCATGCTGATAACGTGTT
pTM3-M1-R	atcgataccgtcgacctcgagCATTGAATGAGTGGATAGTCCATAAGA
pTM3-M2-F	ctatagggcgaattgggtaccTCATCTTCGAAAAGGCATTGG
pTM3-M2-R	atcgataccgtcgacctcgagTCACAAGTGCCAACAGCCAG
pcycD6,1-M1-F	ctatagggcgaattgggtaccTAGGTGGATTTAAATAGAATGAGCGC
pcycD6,1-M1-R	atcgataccgtcgacctcgagATAAGACATAATGATGAAGAGGCTGTTG
pcycD6;1-M2-F	ctatagggcgaattgggtaccTAAGTGAGATATCGAATACCGAATGAA
pcycD6;1-M2-R	atcgataccgtcgacctcgagGTTCATAGCATAAACTCTGACCTCCTA
pIAA29-M1-F	ctatagggcgaattgggtaccAACCAAAACTCTCAAAGCCATTAAG
pIAA29-M1-R	atcgataccgtcgacctcgagTTTGTGAGCTAACAATCAGCCATC
pIAA29-M2-F	ctatagggcgaattgggtaccGATGGCTGATTGTTAGCTCACAAA
pIAA29-M2-R	atcgataccgtcgacctcgagTTCAACGTCAACAGTCAACACCC
pIAA29-M3-F	ctatagggcgaattgggtaccTGAATAATACGTACTCACTACTCGGTAACA
pIAA29-M3-R	atcgataccgtcgacctcgagTGTTGAAATACAAATTTATTTTAGGCTAA
pIAA29-M4-F	ctatagggcgaattgggtaccCATTCAAGTTGGTTGAGTAACTGTTTAA
pIAA29-M4-R	atcgataccgtcgacctcgagCGAATTAATGTGATCCAATTTACATTT
pIAA29-M5-F	ctatagggcgaattgggtaccTGGGTTAGGAAAGGAAAGTAATCAG
pIAA29-M5-R	atcgataccgtcgacctcgagAGTAGCAATGACATAGACACCTTCTACC

Table S8. Primers for EMSA

ID	sequence	
SlMADS48-pGEX4T-1-F	gatctggttccgcgtggatccATGAAGAAAATTGAGGATCCAACAT	
SlMADS48-pGEX4T-1-R	gtcacgatgcggccgctcgagTCAATACATCCATCCCCCACTG	
TM3-Mot1BP-F	ATGAACAACCCTATTTATAGAGTGAGAAAT	5`Biotin
TM3-Mot1BP-R	ATTTCTCACTCTATAAATAGGGTTGTTCAT	5`Biotin
TM3-Mot1mBP-F	ATGAACAACCAAAAAAAAAAAGTGAGAAAT	5`Biotin
TM3-Mot1mBP-R	ATTTCTCACTTTTTTTTTTTGGTTGTTCAT	5`Biotin
TM3-Mot1CP-F	ATGAACAACCCTATTTATAGAGTGAGAAAT	
TM3-Mot1CP-R	ATTTCTCACTCTATAAATAGGGTTGTTCAT	
TM3-Mot2BP-F	ATTTTGAATACTATAAATAGTGATGTAGTT	5`Biotin
TM3-Mot2BP-R	AACTACATCACTATTTATAGTATTCAAAAT	5`Biotin
TM3-Mot2mBP-F	ATTTTGAATAAAAAAAAAAATGATGTAGTT	5`Biotin
TM3-Mot2mBP-R	AACTACATCATTTTTTTTTTTATTCAAAAT	5`Biotin
TM3-Mot2CP-F	ATTTTGAATACTATAAATAGTGATGTAGTT	
TM3-Mot2CP-R	AACTACATCACTATTTATAGTATTCAAAAT	
TM3-Mot3BP-F	CTATGAGAGGCTTAATATAGTAGTATGTTG	5`Biotin
TM3-Mot3BP-R	CAACATACTACTATATTAAGCCTCTCATAG	5`Biotin
TM3-Mot3mBP-F	CTATGAGAGGAAAAAAAAAATAGTATGTTG	5`Biotin
TM3-Mot3mBP-R	CAACATACTATTTTTTTTTTCCTCTCATAG	5`Biotin
TM3-Mot3CP-F	CTATGAGAGGCTTAATATAGTAGTATGTTG	
TM3-Mot3CP-R	CAACATACTACTATATTAAGCCTCTCATAG	
cycD6,1-M2BP-F	ATTAAAGGGTATATACTTTAATTTGGATTTAAGTGTATTT	5`Biotin
cycD6,1-M2BP-R	AAATACACTTAAATCCAAATTAAAGTATATACCCTTTAAT	5`Biotin
cycD6,1-M2mBP-F	ATTAAAGGGTATATAAAAAAAAAAGATTTAAGTGTATTT	5`Biotin
cycD6,1-M2mBP-R	AAATACACTTAAATCTTTTTTTTTTTATATACCCTTTAAT	5`Biotin
cycD6,1-M2CP-F	ATTAAAGGGTATATACTTTAATTTGGATTTAAGTGTATTT	
cycD6,1-M2CP-R	AAATACACTTAAATCCAAATTAAAGTATATACCCTTTAAT	
IAA29-M5BP-F	TTCAAATGCAAATTTCTTATTAAAGTTTGAATTTTAAAAA	5`Biotin
IAA29-M5BP-R	TTTTTAAAATTCAAACTTTAATAAGAAATTTGCATTTGAA	5`Biotin
IAA29-M5mBP-F	TTCAAATGCAAATTTAAAAAAAAAATTTGAATTTTAAAAA	5`Biotin
IAA29-M5mBP-R	TTTTTAAAATTCAAATTTTTTTTTTAAATTTGCATTTGAA	5`Biotin
IAA29-M5CP-F	TTCAAATGCAAATTTCTTATTAAAGTTTGAATTTTAAAAA	
IAA29-M5CP-R	TTTTTAAAATTCAAACTTTAATAAGAAATTTGCATTTGAA	

Table S9. Primers for ChIP-qPCR assay

ID	sequence
qTM3-M1-F	GAGATGTAGATAGAAGAAGAGGAATTTTC
qTM3-M1-R	CATGAACCTTTTTGGATAAGAATGT
qTM3-M2-F	CAATATTGTCTCTTAATTTCTCAAGAAGTC
qTM3-M2-R	GGCTACTTAAGGACACTAGTGACATCA
qIAA29-M5-F	TAGAATGGAGGGAATATAAATTTCATT
qIAA29-M5-R	CCTCCCTCCTGTTGTCATAATTTAT
qcycD6;1-M2-F	AGCATAAGTTTTGTTTTTTAATTGACA
qcycD6;1-M2-R	GCATAAACTCTGACCTCCTAATCAA
